# Long-term Effectiveness of a Peer-Led Asthma Self-management Program on Asthma Outcomes in Adolescents Living in Urban Areas: A Randomized Clinical Trial

**DOI:** 10.1001/jamanetworkopen.2021.37492

**Published:** 2021-12-07

**Authors:** Hyekyun Rhee, Tanzy Love, Mona N. Wicks, Laurene Tumiel-Berhalter, Elizabeth Sloand, Donald Harrington, Leanne Walters

**Affiliations:** 1 School of Nursing, University of Rochester, Rochester, New York; 2 Now with School of Nursing, University of Texas at Austin, Austin; 3 Department of Biostatistics and Computational Biology, University of Rochester Medical Center, Rochester, New York; 4 College of Nursing, University of Tennessee Health Science Center, Memphis; 5 Department of Family Medicine, Jacobs School of Medicine and Biomedical Sciences, State University of New York at Buffalo, Buffalo; 6 School of Nursing, Johns Hopkins University, Baltimore, Maryland; 7 Department of Social Work, Roberts Wesleyan College, Rochester, New York

## Abstract

**Importance:**

Long-term effectiveness of a peer-led asthma self-management program in improving asthma outcomes in adolescents living in urban areas has not been established.

**Objective:**

To determine the long-term effects of a peer-led program on asthma control, quality of life, and asthma management among predominantly racial and ethnic minority adolescents living in urban areas.

**Design, Setting, and Participants:**

A parallel-group, randomized clinical trial was conducted in 2015 to 2019 in 3 metropolitan cities in the US: Buffalo, New York; Baltimore, Maryland; and Memphis, Tennessee. Adolescents aged 12 to 17 years old with persistent asthma were recruited mainly through clinical practices and schools. Participants were followed-up for 15 months after the intervention. Double-blinding was achieved for baseline. Data analysis was performed from June 2019 to June 2020.

**Interventions:**

The intervention group received a peer-led asthma self-management program; the control group received the identical program led by adult health care professionals. Peer leaders made follow-up contacts every other month for 12 months.

**Main Outcomes and Measures:**

The primary outcome was quality of life measured by the Pediatric Asthma Quality of Life Questionnaire, which consists of 3 subscales that measure symptoms (10 items), activity limitations (5 items), and emotional functioning (8 items) in the past 2 weeks. Each item was measured on a 7-point scale, with higher mean scores indicating better quality of life. Secondary measures included the Asthma Control Questionnaire and an asthma self-management index capturing steps to prevent and manage symptoms, self-efficacy, and lung function.

**Results:**

Of 395 eligible adolescents, 35 refused, 38 did not show or were lost to contact, and 2 withdrew before randomization; 320 adolescents participated (mean [SD] age, 14.3 [1.71] years), including 168 boys (52.5%), 251 Black or African American adolescents (78.4%), and 232 adolescents (72.5%) with public health insurance. Of 320 enrolled, 303 were included in the longitudinal analysis. Response rates were 80% or higher at all time points. The peer-led group had greater improvement in outcomes than the adult-led group, with adjusted mean differences (AMDs) between baseline and 15 months of 0.75 vs 0.37 for quality of life (between-group AMD, 0.38; 95% CI, 0.07 to 0.63) and −0.59 vs −0.31 for asthma control (between-group AMD, −0.28; 95% CI, −0.51 to −0.01). Outcomes were not affected by bimonthly contact doses.

**Conclusions and Relevance:**

In this randomized clinical trial, a peer-led asthma self-management education was more effective than an adult-led program in improving asthma outcomes, with the improvements sustained for up to 15 months. These findings suggest that a peer-led asthma self-management program should be considered in addressing the disproportionate asthma burden in racial and ethnic minority adolescents living in urban communities.

**Trial Registration:**

ClinicalTrials.gov Identifier: NCT02293499

## Key Points

**Question:**

Is a peer-led asthma program more effective than an adult-led program in improving asthma outcomes in adolescents living in urban areas?

**Findings:**

In this randomized clinical trial involving 320 predominantly Black or African American adolescents, the peer-led program was significantly more effective than a program led by adult educators in improving asthma control, quality of life, and self-efficacy over time.

**Meaning:**

A peer-led approach may be a compelling alternative to a conventional approach using health care professionals in providing asthma self-management education to racial and ethnic minority adolescents.

## Teaser

This randomized clinical trial examines the long-term effects of a peer-led program on asthma control, quality of life, and asthma management in predominantly racial and ethnic minority adolescents living in urban areas.

## Introduction

Asthma is a leading chronic health condition in adolescents, with 9% of children aged 12 to 17 years in the US reporting a current diagnosis in 2019.^1^ Although efficacious treatment options are available, nearly 60% of US youth with asthma report uncontrolled symptoms.^2^ A disproportionate burden of asthma morbidity among racial and ethnic minority adolescents living in poor urban communities is extensively documented.^3,4,5,6^ Inadequate self-management is one factor associated with high asthma morbidities in urban-residing adolescents.^7,8,9,10,11,12,13,14^ Efforts to improve asthma self-management in urban-residing adolescents are imperative to ameliorate adverse asthma outcomes and promote quality of life.

Learners’ perceived similarities with a person delivering information can increase receptiveness to imparted information, enhancing program outcomes.^15,16,17,18^ Adolescents with chronic conditions tend to seek and heed opinions and guidance from peers similar to themselves.^19,20^ Adolescents with asthma also highly value support from peers with asthma.^21,22,23^ Interactions among adolescents with asthma are positively associated with asthma management^24,25^ and overall well-being.^26,27,28^ Therefore, capitalizing on positive peer dynamics in implementing an asthma self-management program for adolescents appears beneficial.

A peer-led asthma education program was found effective in improving quality of life in school-based studies outside the US,^29,30,31^ but its effect on asthma control remains unknown.^32^ We implemented a peer-led asthma self-management program in a community camp setting and demonstrated its efficacy in improving quality of life compared with an adult-led program, particularly among predominantly racial and ethnic minority adolescents living in urban areas.^33^ These findings require replication in a larger sample of urban-dwelling adolescents, and assessment of long-term sustainability.

This multisite randomized clinical trial examined the long-term effects of a peer-led program on asthma outcomes in adolescents living in urban areas compared with a conventional adult-led approach in 3 US metropolitan communities. We hypothesized that a peer-led program would be more effective than an adult-led program in improving quality of life, asthma control, and asthma self-management over 15 months in adolescents from urban communities.

## Methods

### Study Design

This study reports outcomes of a multisite, parallel-group, randomized clinical trial following the Consolidated Standards of Reporting Trials (CONSORT) reporting guidelines.^34^ The study protocol (see the Trial Protocol in Supplement 1) was reviewed and approved by a coordinating center and 3 study performance sites. Written informed parent or guardian consent and adolescent assent were obtained. No substantial changes were made to methods after trial commencement. No adverse events were reported or identified during the trial.

### Study Settings

The study was conducted in Buffalo, New York; Baltimore, Maryland; and Memphis, Tennessee, between 2015 and 2019. Recruitment took place at various venues, including clinical practices (95 of 320 [29.7%]), schools (72 of 320 [22.5%]), word of mouth (64 of 320 [20.0%]), and flyers or advertisements (33 of 320 [10.3%]); details are reported elsewhere.^35^

### Study Sample

Eligibility criteria included age 12 to 17 years, urban residency, asthma diagnosis for at least 1 year, persistent asthma defined by the Expert Panel Report-3 (EPR-3)^36^ criteria or current use of a controller medication, asthma-related health care utilization in the past 12 months, and English proficiency. Exclusion criteria included parent-reported serious cognitive or mental health conditions that could potentially confound intervention effects or compromise data quality, particularly for self-report data. Peer leaders had similar eligibility as participants except that they were slightly older (aged 16-20 years) and were nominated by adults (eg, teachers or clinicians). They delivered the education program for the intervention group and were not considered participants in this study.

### Sample Size Determination

Power analysis was conducted using the general linear multivariate model with gaussian errors method for repeated measures linear mixed models to determine the sample size.^37^ The longitudinal trajectories of quality of life a primary outcome, and its treatment-by-time interactions based on participant-level randomization, were modeled: the estimated treatment by time interaction coefficient was 0.6 unit, the estimated SD for error was 1.345, and the random-slope was 1.76. Estimated values with α = .05 yielded a total sample size of 276 that would detect time effect between groups with a power of 0.80. We increased the total sample size to 378 to compensate for a possible attrition rate of 27% found in the minority subsample of our previous study.^33^

### Randomization

A randomized block design using sex and age (12-14 years vs 15-17 years) as blocking factors was implemented. A computer-generated random table for each block was used to assign participants into a group for each site. The coordinating center randomized participants for the 3 sites and attempted to conceal group assignments during the allocation process.

### Intervention and Control Treatments

Intervention and control participants attended a day camp where a manualized asthma self-management program was implemented. Participating sites used different venues for the day camp—outdoor camp, university campus, or hospital facilities—as described elsewhere.^38^ The intervention group attended a camp where trained peer leaders delivered the program to a small group of 4 to 8 adolescents. Peer leaders conducted bimonthly contacts occurring every other month using a standardized script for 12 months to provide continuous support and encouragement. Adult leaders (asthma educators or nurse practitioners) led the control group, delivering the identical educational content at the same venue as the intervention group. Research staff conducted bimonthly contacts for 12 months to match for bimonthly attention. Fidelity of the educational program, assessed by an observer using a detailed checklist, was high (94%-97.5%) and was comparable in both groups. The mean (SD) number of successful bimonthly contacts was higher in the control group than in the intervention group (4.60 [1.50] vs 2.60 [2.02] contacts).^39^ Both groups reported high satisfaction with the program, with 92% to 94% positive ratings for all 7 evaluation items.^38^

### Data Collection and Study Measures

Baseline data were collected at either the project office or participant-selected private locations (eg, home). Questionnaire data were collected at baseline, immediately after day camp, and every 3 months for 15 months after the camp. Spirometry data were obtained at camp and 15 months after the camp. Teens’ age, sex, and race and ethnicity were provided by parents or guardians at baseline. Race and ethnicity were assessed as potential covariates and to describe the study sample.

### Primary Outcome: Quality of Life

The Pediatric Asthma Quality of Life Questionnaire consists of 3 subscales that measure symptoms (10 items), activity limitations (5 items), and emotional functioning (8 items) in the past 2 weeks. Each item was measured on a 7-point scale.^40^ Higher mean scores indicated better quality of life. Cronbach α values at baseline were 0.96 (total scale), 0.94 (symptoms), 0.84 (activity limitation), and 0.91 (emotional functioning).

### Secondary Outcomes

#### Asthma Control

The Asthma Control Questionnaire contains 6 items measured on a 5-point scale regarding youth’s activity, asthma symptoms, and controller medication use in the past 2 weeks.^41^ Higher mean scores indicated worse asthma control. Cronbach α at baseline in this study was 0.86. An additional proxy of asthma control, emergency department visit in the past 3 months (yes = 1, no = 0), was obtained.

#### Asthma Self-management

The Asthma Management Index has 3 subscales, including symptom prevention (9 items), symptom management (7 items), and asthma self-efficacy (14 items).^42^ For symptom prevention and self-efficacy subscales, high mean scores indicated greater regularity of preventive steps (range, 1-3) and self-efficacy (range, 1-6). The total number of actions (range, 0-7) taken to manage symptoms was computed for symptom management. The scale’s validity for measuring self-management in adolescents has been established.^42^ Cronbach α values at baseline were 0.79 (symptom prevention), 0.56 (symptom management), and 0.84 (self-efficacy).

#### Lung Function

Forced expiratory volume in the first second of expiration (FEV_1_) and forced vital capacity (FVC) in liters and the percentage of FVC exhaled in the first second (FEV_1_/FVC) were measured following the American Thoracic Society and European Respiratory Society standardization.^43^ Measurements were taken using a portable spirometer (KoKo Sx1000; nSpire Health) at camp (time 2) and at 15-month follow up (time 7).

### Blinding

Both participants and data collectors were blinded after assignment to intervention at enrollment. Because the camp was held on a different date for each group, participants were informed of the camp date later via telephone and mail without specifically mentioning the treatment group. Because nearly 83% of follow-up questionnaires were completed at home either electronically or by mailing, the blinding of data collectors is inapplicable. Given the overt nature of the intervention, blinding researchers or participants to group assignment at or after the camp program was unattainable, particularly for data collected in person at camp and study exit.

### Statistical Analysis

All outcome measures were measured at the continuous level and were fit for a linear mixed-effects model with an interaction between treatment group and time effects. Interaction terms between treatment group and sex and age (12-14 or 15-17 years) were added to these models. Significant interaction effects are reported. For mixed-effects models, the significance of a term was calculated by the change in explained deviance. For final models, estimates and SEs were calculated along with 2-sided *P* values from the *F* test for the treatment effect, each time point effect, and the interaction effects. The significance threshold for each test is *P* < .05. Sociodemographic variables and asthma-related factors presented in Table 1, along with study sites and season, were initially considered covariates. Of those, only season, site, and sex were associated with 1 or more outcomes measures and, thus, were adjusted for in the final models. For each model, we report the adjusted mean difference (AMD) for each time point compared with baseline for each group and the 95% CI for the difference. Outliers, if any, were examined in the residual analysis, but none were removed. We did not identify substantial departures from the model assumptions. Intention-to-treat analyses were conducted with participants who completed at least 1 follow-up data point. We compared these results with sensitivity analyses that included only those with complete cases. Given no widespread differences in findings, this article reports results from the intention-to-treat analyses. In addition to the main effect of treatment groups, we fit models for moderation of the treatment effect by sex, age, or bimonthly contact dose. Data were analyzed with R statistical software version 3.6.2 (R Project for Statistical Computing). Data analysis was performed from June 2019 to June 2020.

**Table 1.** Participant Characteristics and Study Variables at Baseline, by Treatment Group

Characteristic	Participants, No. (%)		
	Total (N = 320)	Intervention (n = 168)	Control (n = 152)
Age, mean (SD) [range], y	14.27 (1.71) [12-17]	14.33 (1.75) [12-17]	14.19 (1.65) [12-17]
Sex			
Female	152 (47.5)	78 (46.4)	74 (48.7)
Male	168 (52.5)	90 (53.6)	78 (51.3)
Black or African American race	251 (78.4)	131 (77.9)	120 (78.9)
Public health insurance	232 (72.5)	115 (68.5)	117 (77.0)
Age at diagnosis, mean (SD) [range], y	4.11 (4.23) [0-16]	3.98 (4.25) [0-15]	4.23 (4.23) [0-16]
Family history of asthma	203 (63.4)	115 (68.5)	88 (57.9)
Missed controller doses in past 2 wk, median (range), No.	2 (0-14)	2 (0-14)	2 (0-14)
Comorbid conditions			
Food allergy	87 (27.2)	45 (26.8)	42 (27.6)
Eczema	144 (45)	77 (45.8)	67 (44.1)
Allergic rhinitis	153 (47.8)	80 (47.6)	73 (48)
Chronic bronchitis	15 (4.7)	7 (4.2)	8 (5.3)
Quality of life scores, mean (SD) [range]			
Overall	5.18 (1.35) [1.52-7.00]	5.21 (1.38) [1.57-7.00]	5.15 (1.31) [1.52-7.00]
Symptoms	5.01 (1.43) [1.10-7.00]	5.02 (1.47) [1.30-7.00]	4.99 (1.40) [1.10-7.00]
Emotional function	5.42 (1.42) [1.38-7.00]	5.45 (1.45) [1.38-7.00]	5.39 (1.39) [1.62-7.00]
Activity limitation	5.13 (1.38) [1.40-7.00]	5.19 (1.40) [1.40-7.00]	5.07 (1.37) [1.6-7.00]
Asthma control score	1.49 (1.13) [0.00-5.17]	1.48 (1.15) [0.00-5.17]	1.51 (1.10) [0-4.83]
Asthma management index scores, mean (SD) [range]			
Prevention	2.00 (0.47) [1.00-3.00]	1.94 (0.49) [1.00-3.00]	2.07 (0.45) [1.00-3.00]
Management	5.33 (1.54) [0.00-7.00]	5.27 (1.66) [0.00-.007]	5.39 (1.39) [1.00-7.00]
Self-efficacy	4.59 (0.83) [1.71-6.00]	4.63 (0.82) [1.79-6.00]	4.63 (0.83) [1.71-6.00]
Lung function, mean (SD) [range]			
FEV_1_ predicted %	90.37 (18.08) [31.00-132.00]	90.91 (18.06) [40.00-125.00]	89.78 (18.15) [31.00-132.00]
FVC predicted %	97.48 (15.75) [27.00-135.00]	97.30 (15.43) [56.00-135.00]	97.67 (16.14) [27.00-135.00]
FEV_1_/FVC	90.13 (11.94) [45.00-115.00]	90.51 (12.2) [56.00-115.00]	89.73 (11.69) [45.00-111.00]
Healthcare utilization in past 3 mo			
Routine office	206.0 (64.6)	100.0 (59.5)	106.0 (70.2)
Visits, range, No.	0-14	0-14	0-10
Acute office	126.0 (39.5)	63.0 (37.5	63.0 (41.7)
Visits, range, No.	0-12	0-12	0-10
Emergency department	67.0 (20.9)	34.0 (20.2)	33.0 (21.7)
Visits, range, No.	0-20	0-20	0-8
Hospitalization	30.0 (9.4)	17.0 (10.1)	13.0 (8.6)
Visits, range, No.	0-7	0-7	0-6
Specialty	110.0 (34.4)	48 (28.6)	62.0 (40.8)
Visits, range, No.	0-12	0-12	0-10
Missed school days, mean (range), No.	1.29 (0.00-15.00)	1.24 (0.00-15.00)	1.36 (0.00-15.00)

Abbreviations: FEV_1_, forced expiratory volume in 1 second; FVC, forced vital capacity.

## Results

### Sample Characteristics and Descriptions of Study Variables

**Figure 1.**
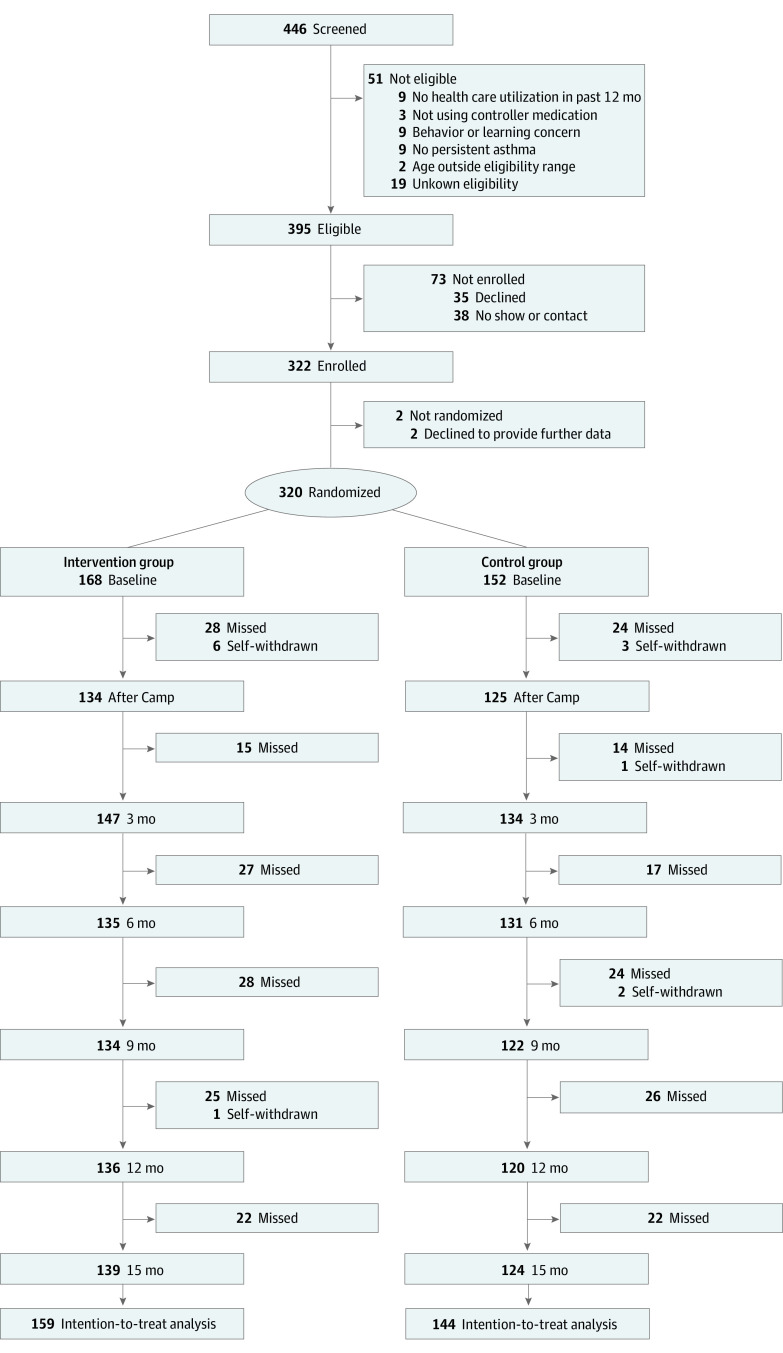
Flow of Participants in the Trial Missed refers to participants who missed data collection at the given time point but still participated.

Figure 1 details participant flow from screening to 15 months after the camp. Of 395 eligible adolescents, 35 refused, 38 did not show or were lost to contact, and 2 withdrew before randomization; in total, 320 adolescents (168 intervention and 152 control) were enrolled, including 131 from Buffalo, New York; 86 from Baltimore, Maryland; and 103 from Memphis, Tennessee. Of those, 17 (5.3%) lost after randomization were considered dropouts (9 intervention and 8 control participants). Reasons for dropouts were unknown as we failed to reach them. A greater percentage of White adolescents discontinued their participation after randomization than racial and ethnic minority adolescents (7 of 44 White participants [15.9%] vs 10 of 276 racial and ethnic minority participants [3.6%]). Dropouts did not appear different from those who remained in the study. Because of the high retention rate, this study has at least 80% power at α = .05 (276 participants were estimated to achieve 80% power).

Sociodemographic characteristics, asthma-related factors, and study variables at baseline are provided in Table 1. The mean (SD) age of the cohort was 14.3 (1.71) years; 168 boys (52.5%), 251 Black or African American adolescents (78.4%), and 232 adolescents (72.5%) with public health insurance were included. Early-onset asthma diagnosed before the age of 6 years was reported by 217 participants (67.8%); 203 (63.4%) reported at least 1 biological family member with asthma. Uncontrolled asthma was reported by 67.0% (214 participants) of the sample, of which 68.0% (146 participants) had very poorly controlled asthma defined by the EPR-3.^36^

eTable 1 in Supplement 2 summarizes the mean (SD) of outcome measures in each group for each time point. Response rates for all measures were approximately 80% or greater at follow-up time points, except for the 15-month spirometry, which had a 67% completion rate (205 participants) compared with 81% (259 participants) at baseline.

### Treatment Effects on Quality of Life (Primary Outcome)

**Figure 2.**
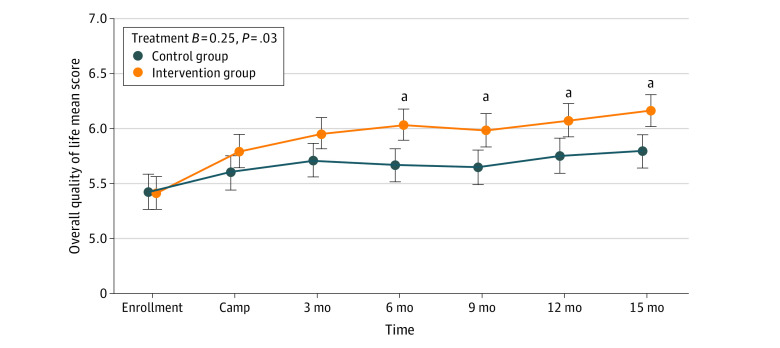
Long-term Patterns of Overall Quality of Life by Group The error bars show 1 SE around the estimate. The effect size of the difference between the groups is given in the key. ^a^Denotes each time point that is different between the treatment groups.

Quality of life improved over 15 months for both groups for all 3 subscales (Table 2). The intervention group showed greater improvement than the control group in overall quality of life (AMD at 15 months, 0.75 vs 0.37; between-group AMD, 0.38; 95% CI, 0.07-0.63) (Figure 2). Similar group differences were found in all 3 subscales (eFigure 1 in Supplement 2). Group differences became more prominent over time in the symptoms subscale for which the intervention group improved steadily from 3 to 15 months while the control group trend plateaued with little change after 3 months (*P* for interaction = .04) (eFigure 1A in Supplement 2). Quality of life was consistently higher among male participants (eFigure 1 in Supplement 2).

**Table 2.** Longitudinal Associations of the Treatment With Outcome Measures in AMDs vs Baseline for Each Group After Controlling for Sex, Camp Season, and Site^a^

Variables and treatment group	Treatment effect size, *B* (95% CI)	*P* value		Adjusted mean score at baseline	AMD (95% CI)											
		Treatment effect	Treatment time interaction		After camp	*P* value	3 mo	*P* value	6 mo	*P* value	9 mo	*P* value	12 mo	*P* value	15 mo	*P* value
Quality of life																
Overall																
Control	0	.03	.09	5.56	0.18 (−0.03 to 0.38)	.08	0.29 (0.08 to 0.50)	.007	0.24 (0.03 to 0.46)	.02	0.23 (0.01 to 0.44)	.03	0.33 (0.12 to 0.54)	.002	0.37 (0.16 to 0.58)	<.001
Intervention	0.25 (0.02 to 0.47)			5.55	0.38 (0.19 to 0.58)	<.001	0.54 (0.34 to 0.74)	<.001	0.62 (0.41 to 0.83)	<.001	0.57 (0.37 to 0.77)	<.001	0.66 (0.46 to 0.86)	<.001	0.75 (0.55 to 0.95)	<.001
Symptoms																
Control	0	.02	.04	5.43	0.19 (−0.04 to 0.41)	.10	0.30 (0.08 to 0.53)	.009	0.25 (0.02 to 0.48)	.03	0.27 (0.04 to 0.50)	.02	0.37 (0.15 to 0.60)	.001	0.38 (0.15 to 0.61)	.001
Intervention	0.27 (0.03 to 0.5)			5.40	0.42 (0.20 to 0.63)	<.001	0.59 (0.37 to 0.80)	<.001	0.68 (0.46 to 0.91)	<.001	0.65 (0.43 to 0.87)	<.001	0.76 (0.54 to 0.97)	<.001	0.85 (0.63 to 1.07)	<.001
Activity limitation																
Control	0	.04	.62	5.44	0.23 (0.01 to 0.45)	.03	0.34 (0.12 to 0.56)	.003	0.22 (−0.02 to 0.45)	.06	0.31 (0.08 to 0.54)	.008	0.38 (0.15 to 0.61)	.001	0.37 (0.14 to 0.60)	.002
Intervention	0.23 (0 to 0.46)			5.51	0.41 (0.19 to 0.62)	<.001	0.46 (0.24 to 0.67)	<.001	0.49 (0.26 to 0.71)	<.001	0.51 (0.29 to 0.72)	<.001	0.54 (0.33 to 0.75)	<.001	0.64 (0.42 to 0.86)	<.001
Emotional function																
Control	0	.05	.12	5.79	0.13 (−0.09 to 0.34)	.24	0.24 (0.02 to 0.46)	.03	0.22 (−0.01 to 0.44)	.05	0.13 (−0.10 to 0.35)	.26	0.25 (0.03 to 0.47)	.02	0.36 (0.14 to 0.58)	.002
Intervention	0.23 (0 to 0.47)			5.76	0.32 (0.12 to 0.53)	.002	0.53 (0.32 to 0.74)	<.001	0.59 (0.37 to 0.81)	<.001	0.51 (0.30 to 0.73)	<.001	0.61 (0.40 to 0.82)	<.001	0.68 (0.47 to 0.89)	<.001
Asthma control																
Control	0	.04	.14	1.19	−0.19 (−0.37 to −0.01)	.04	−0.19 (−0.37 to −0.00)	.04	−0.16 (−0.35 to 0.03)	.10	−0.25 (−0.44 to −0.06)	.01	−0.20 (−0.39 to −0.02)	.03	−0.31 (−0.49 to −0.12)	.001
Intervention	−0.19 (−0.37 to −0.01)			1.21	−0.37 (−0.54 to −0.19)	<.001	−0.41 (−0.59 to −0.24)	<.001	−0.49 (−0.67 to −0.30)	<.001	−0.50 (−0.68 to −0.32)	<.001	−0.52 (−0.70 to −0.35)	<.001	−0.59 (−0.77 to −0.41)	<.001
ED visit (log odds)^b^																
Control	0	.40	.02	−2.48	NA^b^	NA^b^	−0.44 (−0.86 to −0.03)	.03	−0.52 (−0.99 to −0.05)	.03	−0.80 (−1.29 to −0.31)	.001	−0.67 (−1.12 to −0.22)	.004	−0.99 (−1.49 to −0.49)	<.001
Intervention	0.17 (−0.38 to 0.72)			−2.30	NA^b^	NA^b^	−1.23 (−1.67 to −0.80)	<.001	−0.89 (−1.34 to −0.45)	<.001	−1.32 (−1.85 to −0.79)	<.001	−1.65 (−2.23 to −1.06)	<.001	−1.62 (−2.15 to −1.08)	<.001
Asthma self-management																
Symptom prevention																
Control	0	.54	.06	1.97	0.06 (−0.02 to 0.15)	.13	0.03 (−0.06 to 0.11)	.54	−0.01 (−0.10 to 0.08)	.87	0.06 (−0.03 to 0.14)	.20	0.11 (0.03 to 0.20)	.01	0.13 (0.04 to 0.21)	.005
Intervention	−0.03 (−0.12 to 0.06)			1.86	0.17 (0.09 to 0.25)	<.001	0.18 (0.10 to 0.26)	<.001	0.15 (0.07 to 0.24)	<.001	0.17 (0.09 to 0.26)	<.001	0.19 (0.11 to 0.27)	<.001	0.15 (0.07 to 0.24)	<.001
Symptom management																
Control	0	.59	.78	5.09	0.28 (−0.00 to 0.56)	.05	0.23 (−0.06 to 0.51)	.12	0.17 (−0.13 to 0.46)	.26	0.15 (−0.14 to 0.45)	.30	0.15 (−0.14 to 0.44)	.30	0.21 (−0.08 to 0.50)	.16
Intervention	0.06 (−0.15 to 0.27)			5.00	0.35 (0.08 to 0.62)	.01	0.47 (0.19 to 0.74)	<.001	0.40 (0.11 to 0.68)	.007	0.27 (−0.01 to 0.55)	.05	0.31 (0.04 to 0.59)	.02	0.50 (0.22 to 0.78)	<.001
Self-efficacy																
Control	0	.008	.83	4.41	0.05 (−0.11 to 0.22)	.52	0.16 (−0.01 to 0.33)	.06	0.20 (0.03 to 0.37)	.02	0.12 (−0.05 to 0.29)	.15	0.15 (−0.02 to 0.32)	.07	0.22 (0.05 to 0.39)	.01
Intervention	0.19 (0.05 to 0.33)			4.51	0.10 (−0.05 to 0.26)	.20	0.27 (0.11 to 0.43)	<.001	0.37 (0.20 to 0.53)	<.001	0.26 (0.10 to 0.42)	.001	0.27 (0.11 to 0.42)	<.001	0.31 (0.15 to 0.47)	<.001
Lung function^c^																
FEV_1_, L																
Control	0	.27	.69	3.08	NA^d^	NA^d^	NA^d^	NA^d^	NA^d^	NA^d^	NA^d^	NA^d^	NA^d^	NA^d^	0.05 (−0.11 to 0.20)	.54
Intervention	0.07 (−0.06 0.21)			3.14	NA^d^	NA^d^	NA^d^	NA^d^	NA^d^	NA^d^	NA^d^	NA^d^	NA^d^	NA^d^	0.08 (−0.07 to 0.23)	.29
FVC, L																
Control	0	.97	>.99	3.69	NA^d^	NA^d^	NA^d^	NA^d^	NA^d^	NA^d^	NA^d^	NA^d^	NA^d^	NA^d^	0.26 (0.13 to 0.40)	<.001
Intervention	0 (−0.12 to 0.13)			3.70	NA^d^	NA^d^	NA^d^	NA^d^	NA^d^	NA^d^	NA^d^	NA^d^	NA^d^	NA^d^	0.26 (0.13 to 0.40)	<.001
FEV_1_/FVC																
Control	0	.14	.42	0.84	NA^d^	NA^d^	NA^d^	NA^d^	NA^d^	NA^d^	NA^d^	NA^d^	NA^d^	NA^d^	−0.05 (−0.08 to −0.02)	.002
Intervention	0.02 (−0.01 to 0.04)			0.85	NA^d^	NA^d^	NA^d^	NA^d^	NA^d^	NA^d^	NA^d^	NA^d^	NA^d^	NA^d^	−0.04 (−0.07 to −0.01)	.02

Abbreviations: AMD, adjusted mean difference; ED, emergency department; FEV_1_, forced expiratory volume in 1 second; FVC, forced vital capacity; NA, not applicable.

^a^ There were 303 participants in the intention-to-treat analysis.

^b^ ED visit data were not collected at the camp (postcamp only).

^c^ In addition to sex, season, and site, FEV_1_, FVC, and FEV_1_/FVC are also adjusted for height and weight at baseline.

^d^ Spirometry was conducted only 2 times at baseline and 15 months after the intervention; hence, cells before 15 months have no data.

### Treatment Effects on Asthma Control and Self-management

**Figure 3.**
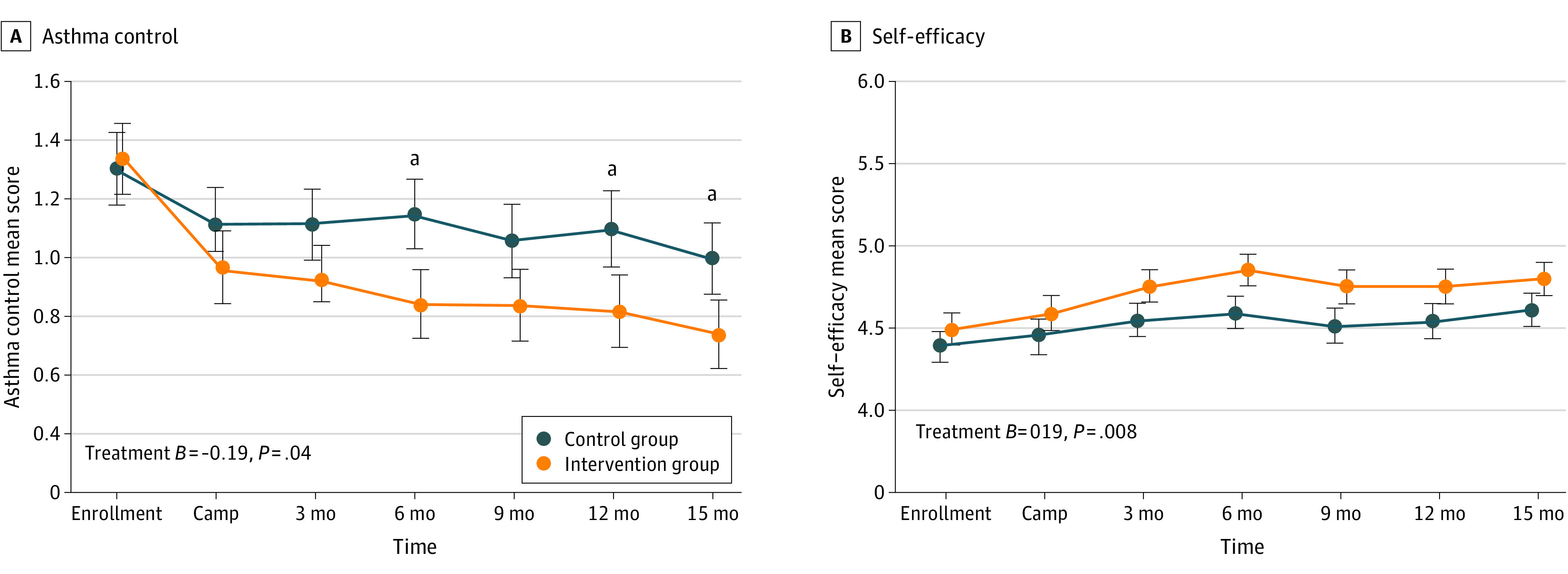
Long-term Patterns of Asthma Control and Self-efficacy by Group The error bars show 1 SE around the estimate. The effect size of the difference between the groups is given in the graph. ^a^Denotes each time point that is different between the treatment groups.

Asthma control improved over time for both groups (Table 2), and the improvement was greater in the intervention group than in the control group (15-month AMD, −0.59 vs −0.31; between-group AMD, −0.28; 95% CI, −0.51 to −0.01) (Figure 3A). In considering individual items of the Asthma Control Questionnaire, group differences were particularly prominent for daily activity, shortness of breath, wheezing and short-acting β-agonist use (eTable 2 in Supplement 2). Male participants had better controlled asthma regardless of group (eFigure 2 in Supplement 2). Both groups were less likely to report emergency department visit after the camp (Table 2).

All 3 subdomains of self-management (symptom prevention, symptom management, and asthma self-efficacy) improved over time, starting at 3 months after camp and sustaining for 15 months for both groups (Table 2). Although both groups improved in symptom prevention and management subdomains, self-efficacy scores in the intervention group were higher than the control group (Figure 3B).

### Treatment Effects on Lung Function (FEV_1_, FVC, and FEV_1_/FVC Ratio)

For both groups, we found little change in FEV_1_ from baseline to 15 months after the intervention, whereas FVC increased, resulting in a decrease in the FEV_1_/FVC ratio in both groups (Table 2). Both groups were similar in the lung function measures.

### Effects of Bimonthly Contacts on Outcome Measures

The control group received more bimonthly contacts than the intervention group, with a mean (SD) number of contacts of 4.60 (1.50) vs 2.60 (2.02). To estimate the effect of bimonthly contacts on outcomes, we created 3 measures: the occurrence of a recent contact (within a month), any contact, and the accumulated number of successful contacts (range, 0-6). Interaction terms between treatment and each measure of contact dose were added to the primary linear mixed-effects models. For the intervention group, each contact measure did not affect the outcomes. However, for the control group, those who had received any contacts had greater improvement in emotional functioning than those who did not receive any contact (*B* = 1.21; 95% CI, 1.25-2.28; *P* = .01) (eFigure 3 in Supplement 2). Treatment outcomes for each group were not affected by recent contact or the total number of contacts.

## Discussion

Consistent with our earlier study,^33^ we found an asthma self-management program led by peer leaders was more effective than one led by health care professionals in improving quality of life, asthma control, and self-efficacy in adolescents living in urban areas. Current findings affirm our earlier efficacy trial of a single site and provide empirical evidence supporting the adaptability of the intervention in various geographical locations and settings. Unlike our earlier efficacy study reporting the program being more effective in male and older adolescents, the current study suggests that intervention effects do not differ by adolescents’ sex or age.

Positive effects of a peer-led program on asthma outcomes have been reported in school-based studies conducted outside the US.^29,30^ Because of a no-to-minimally treated control group, a question remained whether the effects were due to the peer-led approach or educational content the intervention group received. We used an attention control to maximize the comparability between the intervention and control groups in all aspects (education content and venues) to effectively examine the extent to which program leaders, peers vs adults, contribute to any changes in outcomes. Both groups improved for all outcomes including quality of life, asthma control, and asthma management after program implementation. Nonetheless, the effects were greater for the intervention group, suggesting the superiority of a peer-led approach for maximizing the impact of asthma education in adolescents in urban areas. These findings echo those reported in our earlier study^33^ and other studies espousing the superiority of a peer-led approach to those led by adults when delivering health education targeting adolescents.^44^

This study demonstrates the sustainability of program effects for 15 months for both groups. Throughout the study, the intervention group consistently showed greater improvement than the control group for all outcome measures. The 15-month observation period is longer than other studies of a peer-led asthma program including our earlier study for which the follow-up duration was 9 months. Remarkably, program outcomes for both groups were sustained for 15 months along with the group differences. Establishing long-term sustainability of the outcomes of a peer-led program has received little attention. One study^45^ reported that a peer-led program effectively reduced smoking short-term (8 weeks) but failed to demonstrate sustained effect at 1 year.

Our sustained program outcomes for 15 months may have been possible because of the bimonthly contacts conducted for 12 months. Dose-response analyses, however, showed that bimonthly contacts played essentially no role in sustaining program effects for the peer-led group. Despite the substantially lower number of successful bimonthly contacts in the peer-led group than the adult-led group, the peer-led group consistently performed better for most outcomes. The long-term outcomes of our peer-led program, largely independent of periodic follow-up contacts, are compelling, suggesting that the program alone as a free-standing intervention could yield sustainable positive long-term benefits.

Asthma outcomes significantly improved in both groups, supporting beneficial effects of asthma education programs offered to children and adolescents in various formats or venues.^46,47^ EPR-3^36^ maintains that no person with asthma be left without adequate education. Asthma education programs specifically designed for adolescents are scarce,^48^ and their knowledge concerning asthma management is suboptimal,^49,50^ particularly among racial and ethnic minority youth.^14,51^ A peer-led asthma self-management program is a promising approach to asthma education that is effective, sustainable, and developmentally appealing to predominantly racial and ethnic minority, adolescents living in urban communities.

### Limitations

This study has several limitations. First, the nonprobability sampling methods in selecting sites and participants limit generalizability. Caution is warranted in generalizing the findings to urban communities with high percentages of White or Hispanic populations. Second, bimonthly contacts for the intervention group were implemented with low fidelity compared with the control group, although the periodic contacts were not significantly associated with study outcomes. Third, the self-reported study outcomes were susceptible to response bias. Spirometry was conducted only at baseline and 15 months after the intervention. We did not collect other biomarkers to validate or complement the self-reported measure of asthma control. Fourth, the length of follow-up may be too brief to examine the extent to which the intervention affects adolescents’ asthma management as they transition to adulthood, for which a multiyear observation period is necessary.

## Conclusions

Asthma education offered at a day-camp setting was effective in improving asthma outcomes and self-management in primarily minority adolescents living in urban areas, and the positive effects sustained for 15 months. Program effects were greater when led by peer leaders than health care professionals, suggesting that a peer-led approach leveraging adolescents’ high sensitivity to peer influences is a promising strategy to deliver asthma self-management education for racial and ethnic minority adolescents living in urban areas. A peer-led asthma program implemented in venues such as schools, houses of worship, or clinics can improve access to asthma education, ameliorating the disproportionate burden of asthma morbidity in racial and ethnic minority adolescents residing in large US urban communities.

## Notes

Supplement 1.
Trial Protocol

Supplement 2. **eTable 1.** Means and Standard Deviations (SDs) of Study Variables by Treatment Group and Time Point (N=320) **eTable 2.** Longitudinal Effects of the Treatment on Individual Items of the Asthma Control Questionnaire in Adjusted Mean Differences (AMD) in Comparison to Baseline for Each Group After Controlling for Sex, Camp Season and Site (N=303, Intention to Treat Analysis) **eFigure 1.** Long-term Patterns of Quality of Life Subscales for Symptoms (A), Activity Limitations (B), and Emotional Functioning (C) by Group and Sex **eFigure 2.** Long-term Patterns of Asthma Control by Group and Sex **eFigure 3.** Long-term Patterns of Emotional Functioning for Bimonthly Contact Status (Any vs No) by Group

Supplement 3.
Data Sharing Statement

## References

[1] Center for Disease Control and Prevention. Most recent national asthma data. Updated March 30, 2021. Accessed May 5, 2021. https://www.cdc.gov/asthma/most_recent_national_asthma_data.htm

[2] Sullivan PW, Ghushchyan V, Navaratnam P, . National prevalence of poor asthma control and associated outcomes among school-aged children in the United States. J Allergy Clin Immunol Pract. 2018;6(2):536-544.e1. doi:10.1016/j.jaip.2017.06.039 28847656

[3] Milligan KL, Matsui E, Sharma H. Asthma in urban children: epidemiology, environmental risk factors, and the public health domain. Curr Allergy Asthma Rep. 2016;16(4):33. doi:10.1007/s11882-016-0609-6 27026587

[4] Akinbami LJ, Moorman JE, Liu X. Asthma prevalence, health care use, and mortality: United States, 2005-2009. Natl Health Stat Report. 2011;12(32):1-14. 21355352

[5] Akinbami LJ, Moorman JE, Simon AE, Schoendorf KC. Trends in racial disparities for asthma outcomes among children 0 to 17 years, 2001-2010. J Allergy Clin Immunol. 2014;134(3):547-553.e5. doi:10.1016/j.jaci.2014.05.037 25091437 PMC4757843

[6] Poowuttikul P, Saini S, Seth D. Inner-city asthma in children. Clin Rev Allergy Immunol. 2019;56(2):248-268. doi:10.1007/s12016-019-08728-x 30666508

[7] Akinbami LJ, Moorman JE, Garbe PL, Sondik EJ. Status of childhood asthma in the United States, 1980-2007. Pediatrics. 2009;123(3)(suppl):S131-S145. doi:10.1542/peds.2008-2233C 19221156

[8] McQuaid EL, Kopel SJ, Klein RB, Fritz GK. Medication adherence in pediatric asthma: reasoning, responsibility, and behavior. J Pediatr Psychol. 2003;28(5):323-333. doi:10.1093/jpepsy/jsg022 12808009

[9] Kyngäs HA, Kroll T, Duffy ME. Compliance in adolescents with chronic diseases: a review. J Adolesc Health. 2000;26(6):379-388. doi:10.1016/S1054-139X(99)00042-7 10822178

[10] Akinbami LJ, Schoendorf KC. Trends in childhood asthma: prevalence, health care utilization, and mortality. Pediatrics. 2002;110(2, pt 1):315-322. doi:10.1542/peds.110.2.315 12165584

[11] Bauman LJ, Wright E, Leickly FE, . Relationship of adherence to pediatric asthma morbidity among inner-city children. Pediatrics. 2002;110(1, pt 1):e6. doi:10.1542/peds.110.1.e6 12093987

[12] Bellin MH, Newsome A, Land C, . Asthma home management in the inner-city: what can the children teach us? J Pediatr Health Care. 2017;31(3):362-371. doi:10.1016/j.pedhc.2016.11.002 27955875 PMC6407130

[13] Naimi DR, Freedman TG, Ginsburg KR, Bogen D, Rand CS, Apter AJ. Adolescents and asthma: why bother with our meds? J Allergy Clin Immunol. 2009;123(6):1335-1341. doi:10.1016/j.jaci.2009.02.022 19395075 PMC3064885

[14] Bruzzese J-M, Stepney C, Fiorino EK, . Asthma self-management is sub-optimal in urban Hispanic and African American/black early adolescents with uncontrolled persistent asthma. J Asthma. 2012;49(1):90-97. doi:10.3109/02770903.2011.637595 22149141 PMC4515962

[15] Bandura A. Social Foundation of Thoughts and Action. Prentice-Hall, Inc; 1986.

[16] Ozer EJ, Weinstein RS, Maslach C, Siegel D. Adolescent AIDS prevention in context: the impact of peer educator qualities and classroom environments on intervention efficacy. Am J Community Psychol. 1997;25(3):289-323. doi:10.1023/A:1024624610117 9332965

[17] Parcel GS, Simons-Morton BG, Kolbe LJ. Health promotion: integrating organizational change and student learning strategies. Health Educ Q. 1988;15(4):436-450. doi:10.1177/109019818801500405 3230018

[18] Milburn K. A critical review of peer education with young people with special reference to sexual health. Health Educ Res. 1995;10(4):407-420. doi:10.1093/her/10.4.407 10159674

[19] Zimbardo PG, Leippe MR. The Psychology of Attitude Change and Social Influence. McGraw-Hill; 1991.

[20] Sattoe JNT, Jedeloo S, van Staa A. Effective peer-to-peer support for young people with end-stage renal disease: a mixed methods evaluation of Camp COOL. BMC Nephrol. 2013;14:279. doi:10.1186/1471-2369-14-279 24359407 PMC3878094

[21] Kyngäs H. Support network of adolescents with chronic disease: adolescents’ perspective. Nurs Health Sci. 2004;6(4):287-293. doi:10.1111/j.1442-2018.2004.00207.x 15507049

[22] Rhee H, Wenzel J, Steeves RH. Adolescents’ psychosocial experiences living with asthma: a focus group study. J Pediatr Health Care. 2007;21(2):99-107. doi:10.1016/j.pedhc.2006.04.005 17321909

[23] Koster ES, Philbert D, de Vries TW, van Dijk L, Bouvy ML. “I just forget to take it”: asthma self-management needs and preferences in adolescents. J Asthma. 2015;52(8):831-837. doi:10.3109/02770903.2015.1020388 26037397

[24] Cohen R, Franco K, Motlow F, Reznik M, Ozuah PO. Perceptions and attitudes of adolescents with asthma. J Asthma. 2003;40(2):207-211. doi:10.1081/JAS-120017992 12765323

[25] Butler K, Cooper WO. Adherence of pediatric asthma patients with oral corticosteroid prescriptions following pediatric emergency department visit or hospitalization. Pediatr Emerg Care. 2004;20(11):730-735. doi:10.1097/01.pec.0000144914.78124.6f 15502653

[26] Berg J, Tichacek MJ, Theodorakis R. Evaluation of an educational program for adolescents with asthma. J Sch Nurs. 2004;20(1):29-35. doi:10.1177/10598405040200010601 14731108

[27] Velsor-Friedrich B, Vlasses F, Moberley J, Coover L. Talking with teens about asthma management. J Sch Nurs. 2004;20(3):140-148. doi:10.1177/10598405040200030401 15147228

[28] Balfour-Lynn L. Growth and childhood asthma. Arch Dis Child. 1986;61(11):1049-1055. doi:10.1136/adc.61.11.1049 3098185 PMC1778120

[29] Al-sheyab N, Gallagher R, Crisp J, Shah S. Peer-led education for adolescents with asthma in Jordan: a cluster-randomized controlled trial. Pediatrics. 2012;129(1):e106-e112. doi:10.1542/peds.2011-0346 22157137

[30] Shah S, Peat JK, Mazurski EJ, . Effect of peer led programme for asthma education in adolescents: cluster randomised controlled trial. BMJ. 2001;322(7286):583-585. doi:10.1136/bmj.322.7286.583 11238152 PMC26550

[31] Gibson PG, Shah S, Mamoon HA. Peer-led asthma education for adolescents: impact evaluation. J Adolesc Health. 1998;22(1):66-72. doi:10.1016/S1054-139X(97)00203-6 9436069

[32] Kew KM, Carr R, Crossingham I. Lay-led and peer support interventions for adolescents with asthma. Cochrane Database Syst Rev. 2017;4(4):CD012331. doi:10.1002/14651858.CD012331.pub2 28421600 PMC6478329

[33] Rhee H, Belyea MJ, Hunt JF, Brasch J. Effects of a peer-led asthma self-management program for adolescents. Arch Pediatr Adolesc Med. 2011;165(6):513-519. doi:10.1001/archpediatrics.2011.79 21646583 PMC3252732

[34] Schulz KF, Altman DG, Moher D; CONSORT Group. CONSORT 2010 statement: updated guidelines for reporting parallel group randomised trials. BMJ. 2010;340:c332. doi:10.1136/bmj.c332 20332509 PMC2844940

[35] Grape A, Rhee H, Wicks M, Tumiel-Berhalter L, Sloand E. Recruitment and retention strategies for an urban adolescent study: lessons learned from a multi-center study of community-based asthma self-management intervention for adolescents. J Adolesc. 2018;65:123-132. doi:10.1016/j.adolescence.2018.03.004 29587184 PMC5932256

[36] National Asthma Education and Prevention Program. Expert Panel Report 3 (EPR-3): guidelines for the diagnosis and management of asthma—summary report 2007. J Allergy Clin Immunol. 2007;120(5)(suppl):S94-S138. doi:10.1016/j.jaci.2007.09.043 17983880

[37] Kreidler SM, Muller KE, Grunwald GK, . GLIMMPSE: online power computation for linear models with and without a baseline covariate. J Stat Softw. 2013;54(10):1-26. doi:10.18637/jss.v054.i10 PMC3882200 24403868

[38] Grape A, Rhee H, Sanchez P. Evaluation of a peer-led asthma self-management group intervention for urban adolescents. J Pediatr Nurs. 2019;45(March-April):1-6. doi:10.1016/j.pedn.2018.12.011 30594886 PMC6501823

[39] Rhee H, Grape A, Tumiel-Berhalter L, Wicks M, Sloand E, Butz A. Fidelity of a peer-led asthma self-management intervention and its attention control in a multisite study of urban adolescents. Res Nurs Health. 2020;43(2):195-205. doi:10.1002/nur.22001 31793688 PMC7047519

[40] Juniper EF, Guyatt GH, Feeny DH, Ferrie PJ, Griffith LE, Townsend M. Measuring quality of life in children with asthma. Qual Life Res. 1996;5(1):35-46. doi:10.1007/BF00435967 8901365

[41] Juniper EF, Gruffydd-Jones K, Ward S, Svensson K. Asthma Control Questionnaire in children: validation, measurement properties, interpretation. Eur Respir J. 2010;36(6):1410-1416. doi:10.1183/09031936.00117509 20530041

[42] Bruzzese J-M, Sheares BJ, Vincent EJ, . Effects of a school-based intervention for urban adolescents with asthma: a controlled trial. Am J Respir Crit Care Med. 2011;183(8):998-1006. doi:10.1164/rccm.201003-0429OC 21139088 PMC3086747

[43] American Thoracic Society; European Respiratory Society. ATS/ERS recommendations for standardized procedures for the online and offline measurement of exhaled lower respiratory nitric oxide and nasal nitric oxide, 2005. Am J Respir Crit Care Med. 2005;171(8):912-930. doi:10.1164/rccm.200406-710ST 15817806

[44] Mellanby AR, Rees JB, Tripp JH. Peer-led and adult-led school health education: a critical review of available comparative research. Health Educ Res. 2000;15(5):533-545. doi:10.1093/her/15.5.533 11184213

[45] Albrecht SA, Caruthers D, Patrick T, . A randomized controlled trial of a smoking cessation intervention for pregnant adolescents. Nurs Res. 2006;55(6):402-410. doi:10.1097/00006199-200611000-00004 17133147

[46] Guevara JP, Wolf FM, Grum CM, Clark NM. Effects of educational interventions for self management of asthma in children and adolescents: systematic review and meta-analysis. BMJ. 2003;326(7402):1308-1309. doi:10.1136/bmj.326.7402.1308 12805167 PMC161636

[47] Harris K, Kneale D, Lasserson TJ, McDonald VM, Grigg J, Thomas J. School-based self-management interventions for asthma in children and adolescents: a mixed methods systematic review. Cochrane Database Syst Rev. 2019;1(1):CD011651. doi:10.1002/14651858.CD011651.pub2 30687940 PMC6353176

[48] Srof B, Taboas P, Velsor-Friedrich B. Adolescent asthma education programs for teens: review and summary. J Pediatr Health Care. 2012;26(6):418-426. doi:10.1016/j.pedhc.2011.03.010 23099308

[49] Desager K, Vermeulen F, Bodart E. Adherence to asthma treatment in childhood and adolescence: a narrative literature review. Acta Clin Belg. 2018;73(5):348-355. doi:10.1080/17843286.2017.1409684 29228891

[50] Kaplan A, Price D. Treatment adherence in adolescents with asthma. J Asthma Allergy. 2020;13:39-49. doi:10.2147/JAA.S233268 32021311 PMC6969681

[51] Seth D, Saini S, Poowuttikul P. Pediatric inner-city asthma. Pediatr Clin North Am. 2019;66(5):967-979. doi:10.1016/j.pcl.2019.06.012 31466685

