# Supplementary material for: Long-term Effectiveness of a Peer-Led Asthma Self-management Program on Asthma Outcomes in Adolescents Living in Urban Areas: A Randomized Clinical Trial
Source: JAMA Netw Open. 2021 Dec 7;4(12):e2137492. doi: 10.1001/jamanetworkopen.2021.37492 (PMC8652603; doi:10.1001/jamanetworkopen.2021.37492)
Supplement: Supplement 1. — Trial Protocol [file jamanetwopen-e2137492-s001.pdf]

University of Rochester RSRB Protocol

Title – PLASMA: Peer led asthma self-management for adolescents

Principal Investigator: Hyekyun Rhee, PhD, PNP
University of Rochester Medical Center
School of Nursing

## 1. STUDY OVERVIEW – PURPOSE AND BACKGROUND

Asthma, the most common pediatric chronic condition, is a serious problem for many adolescents.[1] In 2010, nearly 11% of adolescents (2.7 million) ages 12 through 17 years in the US reported current asthma.[2] This age cohort suffers greater asthma-related morbidity and mortality than younger children.[3, 4] Asthma disproportionately affects inner-city youth, where asthma severity has increased and achieving optimum asthma control has been elusive.[5] Several factors including poor socioeconomic conditions, life stresses, and environmental triggers have been found to be associated with poorly controlled asthma in inner-city children.[5] Programs targeting asthma in inner-city children have primarily focused on the modification of environmental factors[6] and addressing disparity in healthcare access.[7] Serious adverse outcomes requiring hospitalization, intubations and cardiopulmonary resuscitation are more common in adolescents than in younger children.[8] Moreover, asthma mortality among teens is approximately twice that of younger children.[3] Asthma results in decreased quality of life due to poor sleep quality and limited activity.[4, 9] The impact of asthma on daily activities is substantial: over 50% of teens with asthma reported some degree of activity limitations.[9, 10]

Limited intervention efforts have been directed to address high inner-city asthma morbidity specifically in adolescents by promoting adequate self-management. In adolescents, peers have the greatest influence over behaviors and psychosocial well-being, even in those with chronic illness. Peer support can increase adherence to disease self-management.[11-13] Adolescents with asthma highly value support from peers with asthma.[14, 15] Positive interactions between adolescents with asthma have been found to have positive impact on their asthma management,[16, 17] and overall psychosocial well-being.[13, 18, 19] Therefore, providing a context in which adolescents with asthma can interact with each other can be beneficial in implementing an asthma self-management program for this age group. Given the high prevalence, limited interventions and serious adverse outcomes of asthma and its impact on quality of life in adolescents, it is imperative to implement effective strategies to improve self-management and health outcomes in this population. Building on Dr. Rhee (PI) previous efficacy trial, the overall goal of the proposed study is to evaluate the effectiveness and generalizability of PLASMA, peer-led asthma self-management for adolescents, in improving asthma outcomes in inner-city adolescents from three metropolitan cities in the Northern, Eastern and Southern US with distinctive historical and cultural backgrounds. Multisite studies have been advocated as an effective approach to strengthening external validity as such studies afford the opportunity to assess the extent to which treatment effects are generalizable to different settings.[20-22] Ascertaining generalizability across sites is important as it guides future translation of study findings into policy and practice.[22] This study will also determine long-term sustainability of PLASMA effects and estimate the economic impact of the intervention. This multi-site study is significant in that it will target the understudied population, inner-city adolescents with asthma in three cities in the US, who present serious challenges to optimum asthma management.

Specific study aims are:

1) To evaluate systematically the effectiveness of a peer-led asthma program in inner-city adolescents with persistent asthma.

Hypothesis: We hypothesize that: relative to the control group, the PLASMA group will report greater improvement over time in (H1) quality of life (primary outcome), and (H2) asthma knowledge, attitudes, outcome expectations, self-efficacy, self-management skills, and asthma control, FEV1 (exploratory outcomes). (H3) The post-PLASMA scores on outcome measures will be higher than pre-program scores from both treatment groups.

2) To examine the mediating effects of the secondary outcomes (knowledge, attitudes, outcome expectations, self-efficacy, self-management skills, asthma control, and FEV1) on the primary outcome (quality of life) of the intervention.

3) To examine the moderating effects of personal factors (e.g., age, sex, family support) on primary and secondary outcomes of the intervention.

4) To evaluate the effects of PLASMA on primary and exploratory outcomes in peer leaders (16-20 years).

Hypothesis: We hypothesize that the peer leaders (n=42) will report significant improvement in quality of life (primary outcome) and secondary outcomes (knowledge, attitudes, outcome expectations, self-efficacy, self-management skills, asthma control and FEV1) over the course of 15 months.

5) To determine the economic impact of the intervention. This aim will be accomplished by (5a) measuring the direct healthcare costs and total costs of the PLASMA program, as compared with the control group; (5b) performing net cost analyses for each type of costs; and (5c) estimating cost-effectiveness ratios of the PLASMA group compared with the control.

Hypothesis: We hypothesize that the direct and overall costs per participant in the PLASMA group will be less than costs per participant in the control group, or cost neutral.

2. CHARACTERISTICS OF STUDY POPULATION

2.1 Subject Characteristics

Asthma and its morbidity disproportionately affect minority children of low-income families in inner cities, where asthma severity has increased and poses serious challenges to achieving adequate asthma control. [5] Disparate burdens of asthma and its adverse outcomes among minority children have been consistently documented in the past 20 years. [4, 23-25] In 2010, asthma prevalence in black children under 18 years was twice that of white youths; children in families below the poverty threshold as defined by the US Census Bureau were 1.5 times more likely to report current asthma than those in families at or above 200% of the poverty line. [2] Compared with white youth, black inner-city children are 4 and 3 times more likely to have asthma-related ED visits and hospitalizations respectively. [4] The three cities where the study will take place, Buffalo, NY; Baltimore MD; Memphis TN demonstrate high rates of pediatric asthma, high health care utilization and high morbidity and mortality rates, thus this study will take place in these cities that are most likely to benefit from a proposed intervention.

a) Number of Subjects:

A total of 378 adolescents (12-17 years) and 42 adolescent peer leaders (16-20 years) and their parents will be recruited for this study. The sample size (N=378) is based on the power analysis using a novel method developed by Roy et al. [26] for a 3-level hierarchical longitudinal design based on the program "RMASS." This method has the advantage of incorporating three levels from the sites and repeated-measures from each subject with random-effects of the time trends at both the subject- and site-level, which are assumed to be equivalent in terms of sampling proportions, number of groups and differential attrition rates over time. In this proposed study, quality of life, the primary outcome, was used to estimate the sample size. Using data from the previous study, [27] the longitudinal trajectories of quality of life and its treatment-by-time interactions based on subject-level randomization were modeled: the estimated treatment*time interaction coefficient was 0.6 units, estimated SDs for error was 15, and the random-slope was 1.76. These estimated values with a Type-I error rate (alpha) of .05 yielded a total sample size of 276 that would detect a time trend over seven time points (T1-T7) between groups with a power of

.80. This sample size was estimated on the assumption that the analyses use multi-site hierarchical 3-level linear mixed-effects models. In the earlier study, the attrition rate of the inner-city subsample (n=55) was 27% including those who failed to attend the camp program (n=13, 23%) and those lost to follow-up (n=2, 4%). To compensate for a possible attrition rate of 27%, the total sample size was increased to 378 (126 for each site; 63 each of two groups).

b) Gender and Age of Subjects:

Recruitment will not be limited on gender. Based on epidemiological data, we will focus on adolescents from 12-17 years of age for campers and 16-20 years of age for peer leaders.

Campers/participants will be stratified by gender and age (younger 12-14 years, older 15-17 years), which emerged as influential covariates in the PI's earlier study. Within each of four blocks generated by each combination of these two covariates, each subject will be randomly assigned to either group using a computer-generated randomization table. Stratified randomization will ensure a similar number of subjects representing each block in the two treatment groups, balancing the influence of age and gender. Stratified randomization will be done separately for each site, and centrally coordinated and managed. Within the treatment group, participants will be assigned to subgroups based on their age [younger (12-14 years) and older groups (15-17)] to allow the study team to adjust the levels of difficulty/complexity of program content delivered at the camp. In doing so, we will attempt to enhance participants' understanding of the information covered in the program. In the previous study, we found no differences in study outcomes or camp satisfaction between gender-matched groups and co-ed groups, so subgroups will be gender-mixed.

c) Racial and Ethnic Origin:

Enrollment of participants will not be restricted to any racial or ethnic groups. By recruiting participants from three cities with high prevalence and incidence of pediatric asthma, (again based on epidemiological data) we anticipate the sample demographic to have a higher proportion of minority populations representative of the neighborhoods targeted (Buffalo, Baltimore and Memphis.)

d) Vulnerable Subjects:

This study will include inner city adolescents between ages 12-17 years. This study will evaluate the effectiveness and generalizability of the peer-led asthma self-management program in adolescents in three cities including Buffalo NY, Baltimore MD and Memphis TN. The intervention has proved effective for inner-city adolescents in Rochester, NY.

2.2 Inclusion and Exclusion Criteria

a) Inclusion Criteria:

Eligibility criteria for adolescent participants ("campers") include:

(1) age between 12-17 years;
(2) physician-diagnosed asthma that has required health service use (preventive or acute) within 12 months prior to recruitment;
(3) persistent asthma determined by current use of a control medication <u>or</u> presenting at least one of the following four symptom levels in the past 4 weeks, as defined by the NAEPP guidelines [28]:

- (a) > 2 days/week of daytime symptoms,
- (b) >3-4 times of nighttime awakening,
- (c) >2 days/week of SABA use, or
- (d) any interference with normal activities due to asthma;

(4) We will include those with chronic health conditions except for those with conditions affecting respiratory system, heart disease, pneumonia, etc., and those with moderate to severe cognitive impairments.
(5) primary residence located in the participating inner cities based on zip codes; and
(6) ability to understand spoken and written English.
Eligibility criteria for peer leaders include:
(1) age between 16-20 years;
(2) nomination from school teachers/nurses or healthcare providers for candidates' exemplary asthma self-management, leadership, and emotional intelligence; and
(3) fulfillment of eligibility criteria (2)-(6) prescribed for adolescent participants.

b) Exclusion Criteria:
1) Adolescents who are pregnant or incarcerated at enrollment
2) Have learning disabilities based on reports from teachers or clinicians will be excluded from the study because such conditions can confound the interpretation of findings.
3) Those who have serious health (other than asthma) and emotional preconditions (e.g., severe depression, anxiety disorders, schizophrenia)

## 3. SUBJECT IDENTIFICATION, RECRUITMENT AND CONSENT

### 3.1 Method of Subject Identification and Recruitment:

Participants (peer leaders and camp participants) will be recruited from a variety of settings including clinical practices, community youth organizations, schools and/or churches in the three cities using clinician referrals, recruitment letters, flyers, or newspaper or website ads. Each clinical practice/school/church from which participants will be recruited will be informed of the eligibility criteria for peer leaders, and we will provide these recruitment sites with an information sheet that includes the eligibility criteria for peer leaders and instructs that referring adults would seek permission from the parents of teens (16-17 years) they wish to refer before referral. Referrals can be done by phone, email or written letter. In their referral, referees will provide the study team with the contact numbers of the nominated teens and their parents. Then, the study team will contact the parent/teen to screen for the nominated teen's qualification for enrollment as a peer leader. For older peer leaders (18-20 years), referees are not required to obtain parental permission prior to nomination. Older teens can also contact the study team directly by responding to flyers, or newspaper or website ads to be considered as peer leaders. Study website will be used to inform potential subjects, parents and providers of the study and make it easier for them to contact study personnel for more information, screening or referrals. The website will be hosted in the UR SON's IT server that hosts www.son.rochester.edu. The URLs for the website will be "buffaloteenasthmacamp.com" or "buffaloteenasthmacamp.org" The URLs will be unavailable to the public until the IRB approval. Each site will choose recruitment strategies that are culturally appropriate and effective in its own community. No adolescents <18 years will be allowed to participate in the study without parental permission either as a peer leader or a camper. Although adolescents who are pregnant at the time of enrollment will not be eligible for the study, any participant already enrolled who become pregnant or incarcerated may continue in the study. Any data points missed will be skipped and data collection will continue at the next scheduled data collection time point. For clarification of continued participation of incarcerated participants: those who are incarcerated will be allowed to remain in the study, but no data collection will take place while they are incarcerated. Upon their release, should this be while follow-up measures are still being collected, they will have the option to continue with follow-up study measures or

discontinue. Essentially, they will not automatically be discontinued or withdrawn due to incarceration, their data collection will pause and resume when they are released unless they choose to withdraw.*

3.2 Process of Consent:

Group assignment will be concealed from participants and data collectors during the enrollment phase. To ensure double blinding during enrollment, data collectors will screen subjects for eligibility and then randomization will be conducted by the coordinating center only. The coordinating center staff will not participate in obtaining informed consent or collection of baseline data. During enrollment, data collectors will obtain informed consent and collect baseline data. Randomization and subsequent group assignment is concealed from sites until Enrollment is completed.

a) Informed Parent Permission/Consent. Consent will be obtained in a non-coercive fashion. Prior to obtaining consent, the site coordinator will provide parents/adolescents with detailed information regarding study participation. Parents and adolescents will be informed that their participation is strictly confidential, that they do not have to answer any questions they do not wish to, and they are free to withdraw from the study at any time. The coordinator will solicit and answer any questions. When all questions have been answered, the coordinator will ask the subject a couple of questions pertaining to study procedure and the content of the consent to ensure the subject's understanding. For Spanish-speaking parents, the consent form will be prepared in their language, and an RA who is fluent in Spanish will assist obtaining the consent following the procedure described above. A parent/guardian will be asked to sign two copies of the form, and the coordinator will also sign the consent form. One copy of the form will be kept; one copy will be given to the parent/guardian.

Teen Assent. Assent will be obtained in a similar manner as the informed consent. The assent form will be written using language that is developmentally appropriate. Adolescents will be told that they do not have to answer any questions they do not wish to, and they are free to stop being in the study at any time. Adolescents will be asked to read the assent form silently while the coordinator reads the assent form to them.

Photo permission will be obtained from the parents or teens if they are >18 years to allow the study staff to take pictures of instructional/non-instructional activities taking place in the camp.

b) Vulnerable populations: The coordinator will solicit and answer questions. To make certain of their understanding of the assent consent, adolescents will be asked to explain back what they are being asked to do and clarification will be provided.

## METHODS AND STUDY PROCEDURES

4.1 Study Procedures and Assessments

Design: This study will use a two-group randomized controlled design implemented in three metropolitan cities: Buffalo, NY, Baltimore, MD, and Memphis, TN. The project coordinator and a research assistant in each city who are not involved in direct patient care of teens with asthma will be responsible for recruitment and consent process taking place in each site. Peer leaders will attend a three-day intense training session that will take place prior to camps, lead small-group instructional activities at a one-day camp (PLASMA group) and follow up their group members bimonthly for 15 months. Camp participants from each site (n=126) will be randomly assigned to the peer-led (PLASMA) group or the adult-led (control) group. Upon enrollment, participants will be stratified by gender and age (younger 12-14 years, older 15-17 years), and randomly assigned to either group within each of four blocks generated by the combination of gender and age strata.

Each group will attend a separate day camp where an asthma self-management program will be implemented either by peer leaders (intervention) or healthcare professionals (control). After the camp, the intervention group will receive peer-leader contacts bimonthly for 14 months using phone calls or other communication technologies (e.g., emails, or short-message system (e.g., texting) of teens' choice. Adult instructors (healthcare professionals) will contact the control group bimonthly phone calls for 14 months using similar methods, but different contact topics.

Both groups (intervention and control – more details below) will provide data at enrollment (T1), at camp (T2), and at 3-, 6-, 9-, 12-, and 15-months post-camp (T3-T7).

The intervention: The PLASMA program focuses on providing learning environments conducive to asthma self-management training. We will use adolescents with asthma as leaders to capitalize on peer dynamics, which are often a powerful force driving behavior changes in adolescents. PLASMA leverages teens' receptiveness to peer influences while addressing their desire for independence by offering a teen-governed program format, thus increasing developmental relevance. The Plasma program will be delivered in a camp settings. A camp setting also provides an informal environment in which participants become naturally acquainted and interact with peers with asthma while participating in learning and recreational activities. This setting can offer practical and scientific advantages as well, including allowing investigators to closely monitor multiple small group sessions to ensure fidelity and to provide immediate assistance to peer leaders. We will utilize a well developed and detailed training manual for adolescents ("Let's Talk about Asthma") that addresses four components of asthma self-management in adolescents, including symptom (1) prevention, (2) monitoring and (3) management; and (4) communication/psychosocial empowering. [29] The manual meticulously covers these four areas based on the National Asthma Education and Prevention Program (NAEPP) guidelines [28] and presents information in a way that increases developmental and contextual relevance to inner-city adolescents. Eligible peer leaders will be recruited via referrals or from local churches or colleges using newspaper ads or flyers. A total of 42 peer leaders (14 in each site) will be trained using a structured asthma self-management manual developed by the study team. Training strategies will involve didactic sessions, discussion, demonstrations, and role-play. A certified asthma educator, who will be trained by the PI to be the peer leader trainer, will lead a 2-day training program (12 hours total) for all three sites. Our previous study demonstrated the adequacy of the format, length and frequency of the training sessions to cover essential training components. Training content includes: <u>Day 1:</u> Asthma basics and prevention and Asthma monitoring and management; <u>Day 2:</u> Communication/ psychosocial issue management/leadership training/hands-on practice in simulated peer-led group settings (role-play). Peer leader training will be offered no earlier than one month prior to the scheduled peer-led camp to maximize retention of acquired information and skills. Peer leaders will be evaluated using a combination of oral and written tests at the end of each session to ensure mastery and proficiency of covered content. If a trainee is absent from a session, she or he will attend an individual make-up session; anyone missing one full day of training will be disqualified. In our earlier study, [30] peer leaders' knowledge, self-efficacy and other asthma outcomes showed declining trends 6-months post-camp, suggesting the need for a booster training session. Booster training has been recommended due to its proven benefits for peer leaders. [31, 32] The peer leader trainer will offer a half-day booster session at 6-months post-camp at each site. To increase peer leader retention, voluntary meetings with research assistants will be offered to the peer leaders after camp and at six months after camp to review next steps of the study and provide an opportunity to socialize. In addition, a bi-monthly newsletter will be designed to include study updates and tips for follow-up phone calls to peers.

Peer-led asthma camps: PLASMA will be implemented in small groups at a camp setting where paired peer-leaders will facilitate learning activities. The research team will oversee the group activities and provide assistance to peer leaders and campers as needed. Locations for camps will be selected based on their convenience for the majority of participants and community-based sites. Campers will be assigned to small groups of 8-10 teens, younger groups (12-14 years) and older groups (15-17). The number of

groups will be based on enrollment at the time of camp and the capacity of the camp facility at each study site with the goal of having an equal number of younger and older groups. Younger leaders (13-17) will facilitate the younger groups, older leaders (18-20) the older groups. In the previous study, we found no differences in study outcomes or camp satisfaction between gender-matched groups and co-ed groups, so groups will be gender-mixed for simplicity in grouping. Group learning activities will closely align with the program manual (LTAA) that consists of three sessions: 1-Asthma basics and prevention; 2- Symptom monitoring and management; and 3-Communication and psychosocial issue management. Each session will last 45-75 minutes, and peer leaders will deliver the content and facilitate participant interactions and strategic thinking. Participants will also learn and practice skills in using the peak flow meter, spacer and inhaler, daily symptom diary and asthma action plan. The groups will compete in a game, "Asthma Jeopardy," to review and apply acquired self-management information and skills in problem-solving. The younger and older groups will play separately. Besides instructional activities, participants will engage in recreational activities that each camp site can accommodate, such as dancing, arts-and-crafts, swimming, or rock climbing.

Peer leader follow-ups: Long-term effects of self-management training can be reinforced by periodic follow-ups and continuous encouragement.[33, 34] Because the effects of asthma programs tend to erode over time, follow-ups after the program have been recommended.[35-37] In this study, peer leaders will follow up with their group members bimonthly, offering continuous peer support and encouragement. Peer leaders will use a contact checklist and script to guide and standardize their follow-up contacts. In our previous study, peer leaders found monthly contacts too frequent, and less than 50% were reached for monthly phone contacts, so here peer follow-ups will take place every two months using multiple contact methods such as emails, and text messaging in addition to phone calls. For peer contact months coinciding with data collection months (i.e., 6- and 12-months post-camp), data collections will be done prior to peer contact. Study team will utilize data management system, REDCap, to monitor peer contact. Timely feedback and assistance to peer leaders will be provided. To reinforce peer contacts, study team will award points for successful contacts, which become the basis for reward at the end of the study. For instance, peer leaders who successfully conduct >80% of peer contacts bimonthly will earn 10 points, with 70 points being the maximum possible points (10 points*7 bimonthly contacts). Gift cards loaded with varying amounts of money (a maximum of $50) will be offered based on accumulated points.

Adult Led Asthma Self-Management (Control Group): The control group will attend a similar camp to the PLASMA group, which will take place within 2 weeks of the peer-led camp to minimize the history effect. Two local healthcare professionals selected by the site-PI will attend peer-leader training sessions to become familiar with the program content, then lead instructional activities. As in peer-led PLASMA, adult leaders will base their instruction on the program manual to ensure comparable program content. Campers will be divided into two age sub-groups (12-14, 15-17 years) for age-appropriate delivery of content. Adult leaders will adopt mainly a didactic format and skill demonstration. Each camper will receive the program manual. After the instructional sessions, campers will participate in "Asthma Jeopardy" and comparable recreational activities. To control for attention, the research assistants will offer bimonthly contacts via similar contact and data entry methods to peer contacts, and will discuss topics unrelated to asthma. The RAs will use a contact checklist and script to guide and standardize their follow-up contacts. The study team will determine the non-asthma topics for each bimonthly follow-up to standardize interactions with participants.

Intervention Fidelity: In a multisite study, ensuring consistent delivery of a program as planned across sites is of paramount importance to reach valid conclusions. PLASMA comprises three components: (1) peer leader training and a booster session at 6-months post-camp, (2) peer-led camps, and (3) bimonthly peer follow-ups. For each component, treatment content and pace will be evaluated using a rating scale.[38] (1) Peer leader (PL) training: The PL trainer, trained by the PI at the study center, will conduct PL training for all three sites based on a manualized curriculum. The PL trainer's adherence to the training manual for each site will be evaluated by the site-PI, who will attend each training session, using

a 3- point fidelity rating scale (1=delivered, 2=deviated (for inaccurate content), and 3=missed). The scale will also allow observers to assess time duration for each delivered content (1=adequate, 2=too short, 3=too long) based on the length of time recommended by the study team. The rating scales submitted by the PI and site PIs will be compared. The study team will revisit any items presenting notable discrepancies until consensus is reached. A minimum of 90% adherence will be required to assure treatment fidelity.

(2) Peer-led camp: The camp program will be guided by the standardized manual used for PL training. Peer leaders' adherence to program content and pace will be assessed. The research team will evaluate each session using a fidelity rating scale similar to that of PL training. A research team member will independently rate the extent of PLs' adherence to the program manual using the fidelity rating scale. Ninety percent adherence will be required to assure treatment fidelity. Campers will also complete a checklist after each session to indicate covered training content and adequacy to assess fidelity from a participant's perspective. (3) Bimonthly peer contacts: PLs will be required to record the date, time and duration of each contact, the method of contact and the number of attempts made for contact. PLs will use a checklist provided by the researchers to structure and standardize their interactions with participants. The contact information and completed checklists for each bimonthly contact will be recorded into the data management system via a link sent by the study team or via paper form submitted to the study team, who will evaluate peer contact fidelity. PLs with inadequate adherence to the contact protocol (e.g., incomplete checklist, delivery of <80% of content listed on the checklist) will be notified and counseled to ensure their adherence for subsequent peer contacts.

Procedures for control treatment fidelity: The study team will provide adult leaders with specific written guidelines that detail the elements to be covered and time lengths for each element. The PI and site-PIs will attend instructional sessions and independently rate the adult leaders' adherence to the guidelines using the 3-point fidelity rating scale. Ratings from the raters will be compared, and any discrepancies reconciled using the same procedure as the PLASMA program. Ninety percent adherence will be required to ensure treatment fidelity. Campers will also complete a checklist after each session to indicate covered training content and adequacy to assess fidelity from a participant's perspective. The RA, like peer leaders, will use a checklist for the bimonthly contacts and contact information utilizing the data management system. The checklist will be evaluated periodically by study team to assure fidelity. Any RA with inadequate adherence to the contact protocol (e.g., incomplete checklist, <80% of successful contacts) will be notified and counseled to ensure her/his adherence for subsequent contacts.

Procedure for subjects who do not attend camp: Enrolled campers who do not attend a camp session, will remain in the study as subjects. These subjects will remain in their assigned intervention group. Follow-up surveys will be sent as scheduled after their assigned intervention group camp occurs. Subjects who do not attend camp will not receive intervention related bi-monthly phone contacts.

Procedure for subject retention post camp: To increase subject retention each site may design strategies for retention appropriate for their site population. These activities could include holding a raffle for survey completion, sending out a newsletter with tips on asthma management and reminders for data collection, inviting subjects to the study office for a meet and greet at the final data collection time point. Each site will obtain approval from their site IRB for any proposed retention activities to be implemented.

Involvement of Primary Care Providers (PCPs): The effects of asthma self-management in controlling and reducing the burdens of the disease can be augmented when self-management activities are coordinated in collaboration with a healthcare provider. Having recognized the important roles that the healthcare providers play in adolescents' asthma self-management, we will attempt to incorporate the clinical system in our programs. For both groups, each subject's primary care provider (PCP) will be informed of the teen patient' study participation and training content in an introductory letter which will be either mailed or faxed to the office. This letter will include general

information about the study, the website link to read more about the camps, and contact information should they want more information. The PCPs will also receive written bi-annual reports from the study team that summarizes the levels of asthma control and medication adherence of their teen patients in the past 6 months. In addition, we will communicate to the PCPs via letter or phone call when research data (e.g., FEV1 < 60% predicted, uncontrolled symptoms, consistent medication non-adherence, recent ED visit) warrant clinical attention. For teens that are experiencing clinically significant symptoms, based on NHBI definition of persistent symptoms we will send a letter to notify the PCP. Letters to PCP's will differ based on whether or not the teen started the study on a controller medication. This will ensure that we are highlighting the increased symptoms as well as current maintenance medication use as reported by the teen. The timing and content of such reports provided to the PCPs (except for the introductory letter) will be documented and taken into consideration in analyzing and interpreting research data. Subjects will be encouraged to communicate with their PCP about their asthma conditions learned through symptom monitoring (e.g., symptom diary, PEFR) during their scheduled or unscheduled office visits.

Measures: Sources of research materials will include standardized questionnaires, demographic form, peak flow meters, spirometry, CMS/TennCare database and medical records. Data from these sources will be collected with parents'/participants' consent and according to the standard procedure approved by the IRB. All data will be used for research purposes only and will be kept confidential and anonymous by using subject IDs without identifiers. Only the PI and authorized research staff will have access to these data.

Screening Measures:

We will use two different screening checklists to identify eligible teens for peer leaders and regular campers. The Peer-leader and camper screening checklists are a tool that will ensure that the peer leaders and the campers are eligible to be part of study.

**Primary Outcome:**

**The Pediatric Asthma Quality of Life Questionnaire (PAQOL)**, a 23-item instrument,[39] consists of three subdomains: activity limitation (5 items), emotional function (8 items), and symptoms (10 items). Higher scores indicate better levels of functioning. This scale has proved a valid and reliable measure of asthma-specific quality of life in adolescents.[39, 40] Longitudinal and cross-sectional construct validity of the scale has been supported.[41] Cronbach's alphas of activity, emotion, and symptom subscales in our earlier study were .84, .93 and .95, respectively.[42]

**Exploratory Outcomes:**

**(a) Adolescent Asthma Knowledge Questionnaire (AAK):** This 30-item instrument is a modification of the original 27-item questionnaire[43] measuring children's knowledge of triggers and symptom identification and asthma management procedures. Cronbach's alpha (α) in the PI's previous study was .62.[42]

**(b) Attitude Toward Asthma Scale (ATA):** This 13-item scale measures children's attitudes toward their asthma on a 5-point Likert-type scale.[44] Sound psychometric properties were demonstrated in a study using 136 children with asthma. Construct validity was supported, and Cronbach's α in the PI's previous study was .85.[45]

**(c) Asthma Self-Efficacy (ASE):** This 14-item scale measures a child's confidence in attack prevention (e.g., learning asthma self-management skills, correct use of medication) and attack management (e.g., control symptoms, decide which medication to use).[46] Evidence of construct validity was demonstrated,[46] and Cronbach's α was .83 in our earlier study.[42]

**(d) Asthma Outcome Expectation Scale (AOE)** measures a construct of “outcome expectations” derived from social cognitive theory. [47] This 15-item scale, developed for caregivers of children with asthma, showed acceptable reliability (α=.69-.72) and validity. [47] It will be revised for use with teens.

(e) **Asthma Medication Changes:** assessments will be done on any changes in medications between contacts by asking the teens at each timepoint if they have been prescribed any new asthma medication or had a change in dose prescribed.

**(f) Asthma Self-Management Skills (ASM):** This instrument includes three self-management indices for adolescents including symptom prevention (11 items), symptom management (9 items), and asthma self-efficacy (14 items)[48]; in a recent high school study, the Cronbach’s α of each subscale was 0.71, 0.67, and 0.84 respectively. [49] Validity of the scale was established. [48]

**(g) Medication Adherence-Self Report (AD-S):** To assess medication adherence, we will use the Horne’s Medication adherence report scale, and also ask teens to report doses missed in the prior 2 weeks. These self-report measures will be administered for each assessment point.

**(h) Asthma Control Questionnaire (ACQ)** is a 6 item standardized clinical scale that assesses asthma control based on the frequency of daytime and night time symptoms, degree of activity limitation and use of rescue medication in the past week.

**(i) Asthma Exacerbation Checklist (AEC):** This will assess the indicators of exacerbation including (1) systemic corticosteroids for asthma for at least 3 days; (2) asthma-specific hospital admission; and (3) asthma-specific ED visits. The self-report asthma exacerbation checklist will be used for all the assessment time points.

**(j) Peak Expiratory Flow Rate (PEFR):** we will provide a manual peak flow meter with training on its use and instruct the teens to use it daily and record the values in a log as we proposed in the application, because this is part of self-management routines that we will attempt to reinforce through our training programs.

**(k) Spirometry** will be performed using a portable KoKo® spirometer (Pulmonary Data Service; Louisville, CO). Predicted values will be based on the equations of Polgar. The primary variables will be forced vital capacity (FVC) and FEV1, to be measured twice, at the camp and 15-months post-camp. The subjects will have the option to have the spirometry performed at the University of Buffalo prior to camp through a pre-camp visit or at camp. Respiratory therapists at each site will be trained to perform the test in accordance with the ATS/ERS standardization of spirometry [52] prior to conducting the tests, and data will be interpreted in collaboration with a pulmonologist at the study center.

**(l) Blood sample** will be obtained for total eosinophil count that has been identified as important biomarkers of asthma in characterizing study population for clinical trials (Szefler et al., 2011). Subjects will have the opportunity to choose to have venous blood samples obtained prior to camp at the University of Buffalo by a certified phlebotomist or at the camp by a certified phlebotomist. We will use an EDTA tube for CBC with eosinophil count. Labels for each tube will only contain study ID and collection date. Total volume of blood drawn for both tests is about 5mls for eosinophil. Then, the blood specimens will be stored and transferred in a cooler to URMC Labs (Department of Pathology and Laboratory Medicine at the University of Rochester) for analysis within 24 hours. After the analysis, we will aliquot the plasma spun from the EDTA tube into separate aliquot tubes to freeze for storage in the Biobehavoral lab within the School of Nursing at -80 degrees C and potential future biomarker testing. For future use of the sampled blood outside of this study, we will seek permission on the consent/assent form.

**Moderators:**

**(l) Perceived Family Support (PFS):** We found that family support influenced quality of life and asthma control in adolescents.[53] This 20-item scale measures individuals' perception of their family fulfilling their needs for support, information and feedback. Construct and criterion validity was demonstrated.[54] Cronbach's α in our previous study was .85. **(m) Sociodemographic Form (SDF)** will be completed by parents to indicate adolescents' race, age, sex, insurance type, parental education and annual family income. We added a bi- or multi race option to the race category. For the number of people living in the home, we will assess the number of adult and children (<18 years old) separately.

<u>**Table 1: Study measures for each assessment point**</u>

| | PRIMARY OUTCOME | EXPLORATORY OUTCOMES | | | | | | | | | | | MODERATOR | |
|---|---|---|---|---|---|---|---|---|---|---|---|---|---|---|
| | | Cognitive factors | | | | Self-management | | | | Asthma control | | | | |
| | PAQL | AA | AT | AS | AOE | ASM | AD-S | | FEF | ACQ | AEC | FEV1 | SD | PFS |
| **T1** | √ | √ | √ | √ | √ | √ | √ | | | √ | √ | | √ | √ |
| **T2** | √ | √ | √ | √ | √ | √ | √ | | √ | √ | √ | √ | | |
| **T3** | √ | | | √ | √ | √ | √ | | √ | √ | √ | | | |
| **T4** | √ | √ | √ | | | √ | √ | | √ | √ | √ | | | |
| **T5** | √ | | | √ | √ | √ | √ | | √ | √ | √ | | | |
| **T6** | √ | √ | √ | | | √ | √ | | √ | √ | √ | | | |
| **T7** | √ | √ | √ | √ | √ | √ | √ | | √ | √ | √ | √ | | |

**Asthma-related Information Forms:**

We will assess the family history of ashtma using the Asthma Family Information Form. In addition, we will collect detailed data on current ashtma medication using the Asthma Medication Form, and any device use including a peak flow meter, spacer or nebulizer using the Asthma Management Device Form.

**Measures for Cost-Effective Analysis**:

Health care utilization and costs associated with asthma will be assessed by (1) Medicaid claims, and (2) medical record reviews. We expect that a majority of participants will be enrolled in Medicaid as 90-95% of inner-city children/adolescents were in previous studies conducted in Rochester, Baltimore and Memphis. The Medicaid claims (to be purchased from the Centers for Medicaid & Medicare Services [CMS]: will provide the best source of data for Medicaid-covered services including office visits, ED visits, inpatient care, and prescribed medications. The Medicaid administrative data contain detailed individual-level information on treatment and procedure codes (ICD-9-CM or CPT codes), service dates and expenditures. For adolescents whose services are not available from CMS data, detailed data on service use and prescribed medications related to asthma will be collected through medical records from PCPs; HIPAA authorization will be obtained from parents. In all sites, medical record review was approved by supporting practices for participants lacking CMS data. In both cases, data will be collected between 3 months before enrollment and 3 months after completion of the 15-month follow-up. Atbaseline and at three month intervals, teens and parents will report the number of missed school days due to asthma and the number of hours missed from parents' gainful employment due to the teen's asthma in the past 3 months.

In addition to the medical record review, participants will complete the Health Care Utilization form (self-report)at baseline and at three month intervals, in which they will indicate the ED visits, hospitalization and office visits in the past 3 months. We will also collect the Asthma Care Related Costs form to assess out-of-pocket and indirect costs associated with asthma care including transportation, child-care and time-off from work.

**Measures of Program Evaluation.**

**(a) Camp Program Evaluation** (**CPE**: 7 items) will be administered at the camp to assess the campers' perception about and overall satisfaction with the program.

**(b) Overall Program Evaluation** (**OPE**: 6 items) will be completed at 15-months follow-up to assess participants' overall perceptions about the study. Each item will be examined individually.

**(c) Peer Leaders Rating Scale** (**PLR**: 8 items)[55], to be administered at the camp, will measure PLASMA teens' perceptions of peer leaders' characteristics (i.e., warmth, expertise, credibility) and "perceived similarity." Cronbach's α of the revised scale was .75 in our prior study. We will also examine items individually.

**(d) Perceived Peer Leader Support Scale** (**PLS**: 10 items) will be administered at 15-months follow-up to measure participants' perceptions of peer-leader support. Cronbach's α was .87 in the earlier study. Each item will be examined individually.

**(e) Overall Program Evaluation by Peer Leaders** (10 items) will be completed by the peer leaders at conclusion of study participation (15-months post camp) to assess their experience and perception about the peer-leader program and its impact on themselves.

4.3 Costs to the Subject

There will be no direct cost to subjects to participate in the study.

4.4 Payment for Participation

Peer leaders will receive up to $550 total: $150 for attending 2 training sessions and one booster session (3 sessions at $50 each); $100 for leading the camp program. Peer leaders may receive varying additional amounts up to $50 in a gift card form for successfully completing >80% of bimonthly contacts during the study period. For both peer leaders and other adolescent participants, $30 will be provided at each time-point for completing baseline/enrollment, 3-, 6-, 9-, and 12- post-camp data (up to $150 total). To maximize participation and retention, the payment rate will increase to $50 for the camp and 15-month follow-up ($100; totaling $250 for regular/camp participants). Bus fare will be paid to subjects who need the service.

Peer leaders who participate in Cohort 1 and opt to remain in the role of peer leader only for a second Cohort (2), will receive up to $200 total payment ($100 for camp session; $50 booster session; up to $50 for 80% compliance with bi-monthly phone contacts). These peer leaders will re-consent for the role as peer leader only with no other study subject obligations for participation in Cohort 2.

5. SUBJECT WITHDRAWALS

Participants will have the option to opt out or continue in the study if they become pregnant or incarcerated after the intervention as described above. For peer leaders, if they do not complete all training sessions, they will become disqualified. Those disqualified peer leaders, however, can continue their participation as regular teen subjects (campers) if they are 16-17 years, after re-consenting as a camper.

6. REPORTABLE EVENTS

Adverse events (AEs) are defined as physical and psychological discomfort (boredom, fatigue, stress) related to completing multiple questionnaires and participating in the day camp program, intense self-management training/program. AEs will also include physical injuries during recreational activities at the camp. Any breach of confidentiality would also be an adverse event.

These events are believed to be possible but are not automatically expected. Procedures will be implemented in order to prevent and address AEs during the study

Mechanism for Reporting Adverse Effects. The project staff will also periodically inquire about adolescents' reactions or feelings throughout the study. The project staff will report any AEs observed in relation to study participation to the site-PI and PI immediately. Review of adverse events will include: (1) Adverse events in aggregate, by attribution (expected or unexpected) and relationship to study intervention, (2) Whether the study accrual pattern warrants continuation/action, and (3) potential protocol violations. The Center Coordinator will also monitor study safety periodically for research staff's adherence to study procedure and IRB compliance (e.g., informed consent). The PI will continuously evaluate risks associated with research procedure and maintain subject confidentiality through bi-weekly team meetings and bi-weekly team meetings with the active recruiting and data collecting team through conference calls with the project staff in other sites. The center coordinator will assist the PI in reviewing, capturing and reporting any adverse events and unanticipated problems to the IRB and NINR using interactive computer modules in the Clinical Data Management System. The PI will assess any external factors or relevant information that may impact the safety of participants or ethics of the study

The study team will report all Adverse Events (AE) to the PI and the site-PI within 1-2 business days for each event. The PI Review all adverse events reported and make a decision as to whether they are an AE or Serious Adverse Event (SAE). Additionally, decisions related to continued participation status of the subject will be reached within 10 working days by DSMC consensus (for necessary AE's). The PI and site-PI will report SAEs to the University of Rochester RSRB, DSMC, and relevant site IRB within 1-2 business days of each event. AEs will be recorded and reported to the RSRB at the annual review and to the DSMC on a quarterly basis. AEs will be captured via the electronic system available for all three sites; this system will be used to provide the quarterly reporting to DSMC and the annual review reporting to the RSRB.

## 7. RISK/BENEFIT ASSESSMENT

### Risks to Subjects

Potential Risks: This study will present minimal potential risks to subjects. Data will be collected primarily using questionnaires and noninvasive physiological measures (peak flow meters and spirometer). However, a small number of subjects may be stressed by having to complete multiple questionnaires every 3 months and monitor peak flow values daily during study participation. Small amount of blood (about 5mls) will be drawn for total eosinophil count, which may cause some teen's emotional stress and physical discomfort. Due to the intensive nature of peer leader training and the camp education program, subjects may experience either fatigue and/or boredom. In addition, peer leaders may experience emotional distress potentially associated with their dealings with camp participants' psychological and behavioral issues as well as any issues related to the illness during bimonthly contacts. Also, injuries may also occur during participation in camp recreational activities. The likelihood of risks associated with the camp program, however, is minimal given the sufficient number of trained adult volunteers and the study staff who will closely monitor the conditions and the safety of camp participants during the camp.

Psychological/Physical Distress: To avoid worsening of physical or emotional preconditions, we will exclude those who have serious health (other than asthma) and emotional preconditions (e.g., severe depression, anxiety disorders, schizophrenia) as indicated in the eligibility criteria. Adult nominators (clinicians, teachers, etc.) of peer leaders will be informed of this eligibility criterion. For regular participants, the coordinator will confirm these criteria with parents. Adolescents and parents will be assured that, if the adolescent's responses demonstrate a risk to themselves or others, the adolescent's primary care provider or the family will be notified immediately.

Emotional stress and physical discomfort associated with blood drawing.  To minimize physical discomfort, an experienced and skilled phlebotomist will perform the procedure.  We will also carefully monitor teens' emotional stress/anxiety prior to the procedure. Anyone who demonstrates excessive degrees of emotional stress will be excluded from the procedure. For each teen, only one additional attempt will be made, should the initial attempt fail in venipuncture.

This study involves minimal risk to participants as the study procedures are noninvasive in nature and do not require any changes in existing treatment regimens.  Should the research team identify any participant with serious asthma symptoms that have not received proper treatment, we will encourage the subject to seek medical attention immediately or refer the subject to his/her primary care provider if known. The research team will hold weekly multi-site standing meetings to review participants' responses to the intervention and discuss any challenges or issues encountered in working with the participants.

Peer leaders will be trained to identify and immediately report to the study team any unusual, alarming comments or concerning behavior that they noted during bimonthly contacts. The study team will provide peer leaders with examples of reportable comments and behaviors. The study team will closely monitor peer leaders' emotional distress potentially associated with their dealings with camp participants' psychological and behavioral issues as well as issues associated with the illness during bimonthly contacts. Specifically, the peer leader trainer or the site study staff will provide each peer leader with ongoing and regularly scheduled supervision and support through brief informal interviews on the phone upon completion of each bimonthly contact period for early detection of peer leaders' emotional burden. Any detected signs of distress will be reported immediately to the PI and site PI. All study team members will have a protocol to follow in instances of concerns: informing the family and the primary care provider and providing immediate referrals if indicated.  Any questionable conditions occurring during the study will be discussed in-depth during weekly project meetings for referral as well as a decision related to continuing study participation.

The camps in Buffalo will be protected by liability and accident medical insurance provided by the University of Rochester in case of physical injury during camp participation. Other non-UR sites (Baltimore and Memphis) will have subcontract with the UR, so will be subject to their own institutional terms and policies pertaining to research-related injuries. For each site, consent forms will contain specific statements describing how injuries resulted from study participation will be managed by the institution. Adult volunteers (primarily nursing students) will closely monitor and assist camp participants. The ratio of the volunteers and campers will be maintained at 1:6 to facilitate close surveillance. Each camp will be attended by a state-certified health practitioner (MD or NP) who will evaluate or treat asthma symptoms or minor injuries at the camp site. Each camp site will be equipped with medical supplies necessary to manage most medical emergency situations. Moreover, we will identify and contact a healthcare center with an ED facility located near the camp site prior to the scheduled camp date in order to ensure that the facility is prepared to manage any serious urgent health issues (e.g., asthma attack or severe physical injury) that may occur at the camp.

Because the spirometry procedure requires forced expiration, some subjects may experience exacerbation of asthma symptoms. To address promptly this rare undesirable effect, participants will be instructed to bring their rescue medication (e.g., Albuterol) to the camp and the 15-month follow-up. Trained clinicians will be available for assistance if needed. Any subjects with known risk factors for spirometry-induced asthma exacerbation will be excluded from the procedure. These tests will be performed in a room that is equipped with a respiratory emergency treatment kit.

Completing study questionnaires may cause boredom and fatigue in adolescents, although the PI's earlier study indicated that teens ages 13-20 spent less than 20 minutes on average on this task. Subjects both peer leaders and campers may also experience boredom and tiredness as they participate in the lengthy peer leader training or the camp education program. If the subjects express or present signs of boredom or fatigue, they will be allowed to take a short break during the administering of questionnaires. The electronic data capture system (REDCap) will be programmed in a way that it will permit multiple logins before completing questionnaires. Adolescents may also become physically and mentally exhausted during the peer leader training sessions and camp program. The research team will monitor any signs of discomfort shown by participants during peer leader training and the camp activities (instructional or recreational), and will be available to intervene in situations causing discomfort to the campers or peer leaders. Adolescents will be informed in the assent form and verbally that they do not have to respond to questions they do not wish to answer and that they are not obligated to attend the full camp program if they feel tired. In addition, adolescents will be encouraged to express any study-related questions or concerns at the time of contact or afterwards.

Confidentiality: To ensure confidentiality, subjects will be assigned an identification number, and they will be informed that all information will be held in confidence. Hardcopy data will be kept in a locked file cabinet in each site's project office. Subject information connecting subject names with identification numbers will be locked in a separate file drawer in each site-PI's office. Any printed subject information will be kept in the three site-PIs' locked drawers and will be shredded immediately if not used. Subjects will be informed that their parents or guardians will not have access to their responses for any of the measures. Adolescents and parents will also be assured that forms will be destroyed safely upon completion of the study. Parents and adolescents will be informed that only the research team, University IRB members, grant sponsors and auditors from NIH will have access to their responses. All data from any parent or adolescent requesting withdrawal from the study will be destroyed at the time of the request. Only aggregated data without any personal identifiers will be reported in publications or presentations.

Adolescents and parents will be informed that questionnaires will be completed online. The procedures to address privacy and confidentiality associated with online completion of measures will be discussed with subjects and indicated in the consent/assent forms. Certain technical information automatically collected during the visit to that Web site, including the Internet domain and Internet address (IP address), the type of browser and operating system, and the date and time of access, will be stored in log files on the server and will not be made available to parties other than the system administrators at the URMC. This information will only be used for purposes related to troubleshooting system problems. Subjects will be assured that online data will be transmitted in a secure method to a server database in the study center at URMC. To access the online questionnaires, a unique login and password will be assigned and given to each subject at enrollment. Logging in will afford access to completing the questionnaire only. No person who has logged on with different logins and passwords will be able to view any other person's responses to the questionnaires. All answers will be sent directly to the study center (URMC) database each time data are submitted; data will not be transmitted by any other means and cannot be retrieved by another person except the researchers in Rochester. In order to increase security, subjects will be counseled not to share these passwords with others, even those enrolled in the study. Data files will be backed up regularly. Project files and databases associated with the study will only be available to research personnel through the authorization of the PIs. In addition, study reports (such as aggregated data in progress reports) generated by the research team will provide total anonymity because no names or identifying information will be part of such reports. Participants and staff will be apprised of their rights and responsibilities under the Privacy Act of 1974, including penalties for violations. All staff involved with the research project will receive training on

their function, roles, and responsibilities to protect and maintain privacy and confidentiality of research participants, and will complete NIH-approved training in this area.

7.2 Benefits to Subjects.

Although it is not automatically assumed that participants will benefit from study participation, the PI's earlier pilot study indicates potential benefits for participants. We anticipate that peer leaders will benefit from participation in the intensive three-day sessions. Leading the PLASMA program may provide peer leaders with opportunities to increase their knowledge and skills for effective asthma management and to develop and exercise leadership skills. Similarly, regular adolescent participants will have an opportunity to learn asthma self-management and build social networks with peers with the same illness. The camp will also provide participants with opportunities for entertainment and social experiences with asthma peers. The control group will attend an adult-led camp and receive the same information from a healthcare professional. If proven effective in this multi-site study, the PLASMA program can be adopted by healthcare providers or third party payers (e.g., HMO or Medicaid) as a standard care program to address asthma morbidity and mortality among inner-city adolescents and to contain health care costs for this group. Moreover, information gained through this study may benefit other adolescents with asthma who would receive a similar program in the future.

Risk/benefit ratio**.** Risks to participants are reasonable in relation to anticipated benefits, with potential benefits far outweighing the risks. Because issues related to confidentiality will be scrupulously explained and managed, the primary concern is use of time. Anticipated benefits, including the peak flow meter and the program manual to keep, asthma self-management training and study incentives, are believed to be adequate compensation for the adolescents' time. Moreover, peer leaders and participants may experience an altruistic benefit in participating in a study that may benefit other adolescents with asthma in the future.

Self-reported (standardized questionnaires) and physiological (PEFR and Spirometry) data collected longitudinally (21 months) will capture systematic and dynamic processes by which the program modifies adolescents' self-management behaviors and health outcomes. The long-term observation will also allow the investigators to examine the sustainability of the intervention. Inclusion of multiple cognitive factors (knowledge, attitude, self-efficacy, and outcome expectations) as mediators will allow us to demonstrate intricate interrelationships or mechanisms between the intervention and asthma outcomes including asthma control and quality of life in adolescents. This study will also systematically investigate the economic impact (e.g., healthcare utilization, prescription and indirect costs) of the intervention, which will offer invaluable data to policy makers who might consider adopting the intervention as a standard care for teens with asthma. Moreover, this multi-site study involving three cities representing the Northeastern, Eastern and Southern sections of the US will provide evidence of the generalizability of the intervention and its impacts, which could further accelerate the adoption and implementation of the program to address the serious health threats of asthma in inner-city adolescents and to contain economic costs to society.

7.3 Alternatives to Participation

There are no alternative courses of action should the subject elect not to participate in the study.

8. CONFIDENTIALIATY OF DATA AND INFORMATION STORAGE

To ensure confidentiality, subjects will be assigned an identification number, and they will be informed that all information will be held in confidence. Subjects will be informed that their parents or guardians will not have access to their responses for any of the measures. Only two documents, the informed consent form and the subject contact form, will contain participant's identifying information. The informed consent form will be kept separate from the de-identified

subject data, in paper form, accessible only to the essential study team. The contact form, containing the participant's contact information (i.e., address and cell/phone numbers), will be needed by the study coordinator/peer leaders for follow-up visits for data collection.

Hardcopy of data, consent forms, subject contact information, and subject information connecting subject names with identification numbers will be kept at the enrolling site for each subject; Buffalo, NY, Baltimore, MD, or Memphis, TN, respectively. Storage of data at the enrolling sites will be in accordance with each site's approved IRB protocol including locked storage with access only by project staff, and secure disposal of records after approved amount of time after study completion.

Adolescents and parents will also be assured that forms will be destroyed safely three years after completion of the study. Parents and adolescents will be informed that only the research team, University IRB members, grant sponsors and auditors from NIH will have access to their responses. All data from any parent or adolescent requesting withdrawal from the study will be destroyed upon request. Only aggregated data without any personal identifiers will be reported in publications or presentations.

The lead PI (Rhee) will maintain identifiers (subject names) to the study IDs in a locked cabinet in the center coordinator's office at all times. The coordinating center (University of Rochester, School of Nursing) will also retain hardcopies of de-identified biometric data (spirometry, weight/height/waist circumferences and blood results), camp training and practice worksheets, and fidelity check forms from all sites in locked storage in the center coordinator's office.

Labels on the tubes of blood samples will include only study ID and collection date. Analyzed blood samples from Baltimore and Memphis sites will be safely discarded by those sites after analysis. Only a small subset of analyzed blood samples from the Buffalo site will be properly prepared and stored in a freezer in the biobehavioral lab located in the UR School of Nursing. The lab is locked all the time and can be accessed only by authorized personnel. Subject information connecting subject names with identification numbers will be locked in a separate file drawer in coordinating center's office.

Data and communication from each site will be shared with the coordinating center (University of Rochester, School of Nursing) via secure REDCap program and the University of Rochester's secure Box cloud storage service. These files are stored locally on a password-protected secure server maintained by the University of Rochester School of Nursing IT Department.

## 9. RESEARCH INFORMATION IN MEDICAL RECORDS

N/A

## 10. DATA ANALYSIS AND DATA MONITORING

10.1 Planned Statistical Analysis: Analyses will be performed using SAS, and R [56] will be used as analysis software to ensure the validity and reproducibility of the results. We will perform descriptive statistics on each outcome measure to look for abnormality and outliers. If a measure is not normally distributed, transformation will be applied. We will identify and investigate outliers for sources of errors, and conduct preliminary analyses on data distribution and bivariate correlations to determine the appropriate statistical model as well as the final interpretation of the results. We will investigate missing data with pattern analysis for data missing completely at random (MCAR), missing at random (MAR) or missing not at random (MNAR), and use statistical methods appropriate for each type such as maximum likelihood and multiple imputation to impute missing values so full analysis can be performed with sensitivity. We will compute Cronbach's alphas for psychometric measures, and assess construct validity using exploratory or confirmatory factor analysis when appropriate. The PLASMA and control groups will be compared on baseline

data to determine any systematic differences; if any are found, analyses will include the variables as covariates in the model to adjust for the imbalances.

Aim 1: A multi-site hierarchical three-level linear mixed-effects model,[26, 56, 57] where level 1 represents repeated measures, level 2 = subject, and level 3 = site, will be used to analyze treatment differences and treatment-by-time interaction described in the following notation by Hedeker and Gibbons.[57]

Aim 2: The mediating effects will be examined mainly using the mediation methods of Baron & Kenny.[58] When found significant, a multiple mediation analysis[59] procedure will be used to further determine whether the effect of PLASMA on quality of life is mediated jointly by multiple exploratory outcomes.

Aim 3: Moderating effects will be examined by multiple linear models and generalized linear models described in Berridge and Crouchley[56] or Chen and Peace [60] along with the above multi-site hierarchical three-level linear mixed-effects models.

Aim 4: This aim will be tested by applying the multi-site hierarchical two-level linear mixed-effects models described in the analysis for Aim 1, with level 1 representing longitudinal measurements and level 2 sites.

Aim 5: The costs of services received during visits to a physician's office, outpatient department, or ED will be estimated by taking the global relative value unit for each service CPT (Current Procedural Terminology) code multiplied by a standard conversion factor.[61] Hospital charges will be converted to costs using department-specific cost-to-charge ratio.[62] Medications and dosages prescribed will be captured; standard wholesale unit cost from the Pharmaceutical Red Book[63] will be used to estimate medication costs. To calculate lost productivity costs of a family with a sick teen, we will estimate total lost income related to each participant by multiplying total number of work hours missed by a parent's hourly wage. Program costs will be estimated from the actual time peer and adult leaders spent in carrying out the program multiplied by hourly earnings, costs of equipment/ supplies, and overhead costs; program time will not include staff time for research-related tasks. We will estimate the average program cost per participant by study group, and include this estimate as one component of direct healthcare costs. The medical or general Consumer Price Index[64] will be used to inflation-adjust costs to current year prices.[64] For each teen at each time-point, we will calculate total direct costs, direct costs by major category (office visit, hospitalization), indirect lost-productivity costs, and total direct and indirect costs. For withdrawals from follow-up, we will impute missing utilization and cost data assuming a linear trend in use and cost given prior experiences, where we will estimate weighted least square regression on log-transformed cost and obtain smearing-retransformed predictions.[65] For PLASMA and control groups separately, we will calculate the cost difference between each time point and baseline for each teen, assuming (s)) he received "standard care" before treatment. Cost differences for each group will be modeled against follow-up points and individual covariates to estimate the independent impacts of each program on costs relative to standard care.

10.2 Data and Safety Monitoring: The proposed project meets the NIH definition of a clinical trial and thus requires a Data and Safety Monitoring Plan. The Data Safety Monitoring Committee (DSMC) will be established for the independent review of data. This study involves minimal risk because "the probability and magnitude of harm or discomfort anticipated in the research are not greater in and of themselves than those ordinarily encountered in daily life or during the performance of routine physical or psychological examinations or tests" (HHS.gov). Therefore, we will form the DSMC. This independent committee will be charged with reviewing safety and trial progress and providing recommendations for study continuation and modification. The DSMC comprises four PI-appointed members who are investigators and biostatisticians independent of

the study protocol, as required by the NIH. The names and affiliations of the board members are listed below. These individuals are not participating in this project and can provide objective feedback and recommendations to the investigators regarding any issues of data safety and monitoring. All have agreed to serve on the DSMC for this study. The Committee Chair elected by the board members will lead the meetings and submit any reports to the NINR if necessary. The PI will provide written reports to the DSMC on the current status of the trial, interim analyses, adverse events and problems encountered. If there are recommendations for consideration (e.g., changes in sample size, modifying outcomes) or amendments to the study protocol, the PI will provide a summary to the committee for review. The PI will be responsible for disseminating the DSMC recommendations to participating clinical sites and the NINR.

- James McMahon, PhD, Associate Professor, Biostatistician/clinical trial expert, University of Rochester School of Nursing
- Jill Halterman, MD, MPH, Professor, Content/clinical trial expert, University of Rochester Medical Center, Department of Pediatrics
- Elizabeth (Betsy) Tolley, PhD, Professor, Biostatistician, University of Tennessee Health Science Center, Biostatistics & Epidemiology Division, Preventive Medicine Department.
- Maria Trent, MD, MPH, Associate Professor, Clinical trial expert, Division of General Pediatrics and Adolescent Medicine, Johns Hopkins School of Medicine.

The DSMC will meet annually (via video-conference). Each meeting will be divided into an open and closed session. Each meeting will be conducted by Roberts Rule of Order, and written summaries and recommendations will be sent to the PI. Necessary changes to the study protocol will be communicated within 48 hours, and changes will be made expeditiously. The open session will be attended by members of the DSMC and the study team (the PI, site-PIs, and study coordinators from each site). During the session, the PI will present general progress of the study, adverse events, subject accrual, protocol compliance, quality control and timeliness. The closed session will be attended by only the DSMC members, who will discuss data presented during the open session and vote on recommendations.

NOTE: This application includes a clinical trial of a behavioral intervention that has already been registered in ClinicalTrials.gov (NCT01161225) during the PI's earlier study in complying with registration and regulatory guidelines for this designation. The registered study protocol will be updated to reflect the current study design and outcomes.

Investigator Time and Resources

This IRB protocol is in partial fulfillment of Just-in-Time (JIT) notice requirements of the National Institutes of Health, National Institute of Nursing Research, in connection with NIH grant application R01 1R01NR014451 (Rhee, PI). The study will be conducted only on condition of grant award notification and funding, which will ensure sufficient investigator time and resources to conduct the study. Sufficient investigator and institutional resources have been verified by the School of Nursing grants management department and the Associate Dean for Research.

## REFERENCES

1. Morgan WJ, Stern DA, Sherrill DL, et al. Outcome of asthma and wheezing in the first 6 years of life. *Am J Respir Crit Care Med*. 2005;172:1253-1258.

2. Bloom B, Cohen RA, Freeman G. Summary health statistics for U.S. children: National health interview survey, 2010. *Vital Health Stat*. 2011;10(250).

3. Akinbami LJ, Schoendorf KC. Trends in childhood asthma: Prevalence, health care utilization and mortality. *Pediatrics*. 2002;110(2):315-322.

4. Akinbami LJ, Moorman JE, Garbe PL, Sondik EJ. Status of childhood asthma in the united states, 1980–2007. *Pediatrics*. March 2009;123(Supplement 3):S131-S145. doi: 10.1542/peds.2008-2233C.

5. William W. B. The national institutes of allergy and infectious diseases networks on asthma in inner-city children: An approach to improved care. *J Allergy Clin Immunol*. 2010;125(3):529-537. doi: 10.1016/j.jaci.2010.01.036.

6. Busse WW, Mitchell H. Addressing issues of asthma in inner-city children. *J Allergy Clin Immunol*. 2007;119(1):43-49. doi: 10.1016/j.jaci.2006.10.021.

7. Scott L, Morphew T, Bollinger ME, et al. Achieving and maintaining asthma control in inner-city children. *J Allergy Clin Immunol*. 2011;128(1):56-63. doi: 10.1016/j.jaci.2011.03.020.

8. Calmes D, Leake BD, Carlisle DM. Adverse asthma outcomes among children hospitalized with asthma in california. *Pediatrics*. 1998;101(5):845-850. doi: 10.1542/peds.101.5.845.

9. Fuhlbrigge AL, Guilbert T, Spahn J, Peden D, Davis K. The influence of variation in type and pattern of symptoms on assessment in pediatric asthma. *Pediatrics*. 2006;118(2):619-625. doi: 10.1542/peds.2005-2963.

10. Davis KJ, DiSantostefano R, Peden DB. Is johnny wheezing? parent-child agreement in the childhood asthma in america survey. *Pediatr Allergy Immunol*. 2011;22(1-Part-I):31-35. doi: 10.1111/j.1399-3038.2010.01016.x.

11. Kyngas H, Rissanen M. Support as a crucial predictor of good compliance of adolescents with a chronic disease. *J Clin Nurs*. 2001;10(6):767-774.

12. Randolph C, Fraser B. Stressors and concerns in teen asthma. *Curr Probl Pediatr*. 1999;29(3):82-93.

13. Berg J, Tichacek MN, Theodorakis R. Evaluation of an educational program for adolescents with asthma. *J Sch Nurs*. 2004;20(1):29-35.

14. Kyngas H. Support network of adolescents with chronic disease: Adolescents' perspective. *Nurs Health Sci*. 2004;6(4):287-293.

15. Rhee H, Wenzel J, Steeves RH. Adolescents' psychosocial experiences living with asthma: A focus group study. *J Pediatr Health Care*. 2007;21(2):99-107.

16. Cohen R, Franco K, Motlow F, Reznik M, Ozuah PO. Perceptions and attitudes of adolescents with asthma. *J Asthma*. 2003;40(2):207-211.

17. Butler K, Cooper WO. Adherence of pediatric asthma patients with oral corticosteroid prescriptions following pediatric emergency department visit of hospitalization. *Pediatr Emerg Care*. 2004;20(11):730-735.

18. Velsor-Friedrich B, Vlasses F, Moberly J, Coover L. Talking with teens about asthma management. *J Sch Nurs*. 2004;20(3):140-148.

19. Balfour-Lynn L. Growth and childhood asthma. *Arch Dis Child*. 1986;61(11):1049-1055.

20. Shadish WR, Cook TD, Campbell DT. *Experiemental and Quasi-Experimental Designs for Generalized Causal Inference.* Boston, MA: Houghton Mifflin Company; 2002.

21. Ferguson L. External valdity, generalizability and knowledge utilization. *J Nurs Scholarsh*. 2004;36(1):16-22.

22. Laws RA, St.George AB, Rychetnik L, Bauman AE. Diabetes prevention research: A systematic review of external validity in lifestyle interventions. *Am J Prev Med*. 2012;43(2):205-214. doi: 10.1016/j.amepre.2012.04.017.

23. Akinbami LJ, LaFleur BJ, Schoendorf KC. Racial and income disparities in childhood asthma in united states. *Discourse Processes*. 2002;2(5):382-387.

24. Halterman JS, Aligne CA, Auinger P, McBride JT, Szilagyi PG. Inadequate therapy for asthma among children in the united states. *Pediatrics*. 2000;105(1 Pt 3):272-276.

25. Gupta RS, Carrión-Carire V, Weiss KB. The widening black/white gap in asthma hospitalizations and mortality. *J Allergy Clin Immunol*. 2006;117(2):351-358. doi: 10.1016/j.jaci.2005.11.047.

26. Roy A, Bhaumik DK, Aryal S, Gibbons RD. Sample size determination for hierarchical designs with different attrition rates. *Biometrics*. 2007;63:699--707.

27. Rhee H, Belyea MJ, Hunt JF, Brasch J. Effects of a peer-led asthma self-management program for adolescents. *Arch Pediatr Adolesc Med*. 2011;165(6):513-519. doi: 10.1001/archpediatrics.2011.79.

28. National Heart, Lung, and Blood Institute. Expert panel report 3: Guidelines for the diagnosis and management of asthma. 2007.

29. Mammen J, Rhee H. Concept analysis: Asthma self-management in adolescents. *Pediatr Allergy Immunol Pulmonol*. 2012;25(4):180-189.

30. Rhee H, McQuillan B, Belyea MJ. Evaluation of a peer-led asthma self-management program and benefits of the program for adolescent peer leaders. *Respir Care*. 2012;57(12):2082-2089.

31. Maticka-Tyndale E, Barnett JP. Peer-led interventions to reduce HIV risk of youth: A review. *Eval Program Plann*. 2010;33(2):98-112. doi: DOI: 10.1016/j.evalprogplan.2009.07.001.

32. Becker CB, Bull S, Smith LM, Ciao AC. Effects of being a peer-leader in an eating disorder prevention program: Can we further reduce eating disorder risk factors?. *Eat Disord*. 2008;16:444-459.

33. Bruzzese JM, Bonner S, Vincent EJ, et al. Asthma education: The adolescent experience. *Patient Educ Couns*. 2004;55(3):396-406.

34. Gebert N, Hummelink R, Konning J, et al. Efficacy of a self-management program for childhood asthma-a prospective controlled study. *Patient Educ Couns*. 1998;35(3):213-20.

35. Lord A, St. Leger LH, Ridge DT, Elisha D. The value of asthma camps for young people in victoria, australia. *Contemporary Nurse*. 2001;11(2-3):133-141.

36. Meng A, Tiernan K, Bernier MJ, Brooks EG. Lessons from an evaluation of the effectiveness of an asthma day camp. *American Journal of Maternal Child Nursing*. 1998;23(6):300-306.

37. Robinson LD. Pediatric asthma self-management: Current concepts. *Journal of the National Medical Association*. 1999;91(8 Suppl):405-445.

38. Song M, Happ MB, Sandelowski M. Development of a tool to assess fidelity to a psycho-educational intervention. *J Adv Nurs*. 2010;66(3):673-682. doi: 10.1111/j.1365-2648.2009.05216.x.

39. Juniper EF, Guyatt GH, Feeny DH, Ferrie PJ, Griffith LE, Townsend M. Measuring quality of life in children with asthma. *Qual Life Res*. 1996;5(1):35-46.

40. Okelo SO, Wu AW, Krishnann JA, Rand CS, Skinner EA, Diette GB. Emotional quality-of-life and outcomes in adolescents with asthma. *J Pediatr*. 2004;145(4):523-529.

41. Raat H, Bueving HJ, de Jongste JC, Grol MH, Juniper EF, van der Wouden JC. Responsiveness, longitudinal-and cross-sectional construct validity of the pediatric asthma quality of life questionnaire (PAQLQ) in dutch children with asthma. *Qual Life Res*. 2005;14(1):265-272.

42. Rhee H, Belyea MJ, Elward KS. Patterns of asthma control perception in adolescents: Associations with psychosocial functioning. *J Asthma*. 2008;45:600-606.

43. Bartholomew LK, Gold RS, Parcel GS, et al. Watch, discover, think and act: Evaluation of computer-assisted instruction to improve asthma self-management in inner-city children. *Patient Educ Couns*. 2000;39(2-3):269-280.

44. Austin JK, Huberty TJ. Development of the child attitude toward illness scale. *J Pediatr Psychol*. 1993;18(4):467-480.

45. Rhee H, Belyea MJ, Cirzynski S, Brasch J. Barriers to asthma self-management in adolescents: Relationships to psychosocial factors. *Pediatr Pulmonol*. 2009;44(2):183-191.

46. Bursch B, Schwankovsky L, Gilbert J, Zeiger R. Construction and validation of four childhood asthma self-management scales: Parent barriers, child and parent self-efficacy, and parent belief in treatment efficacy. *J Asthma*. 1999;36(1):115-128.

47. Holden G, Wade SL, Mitchell H, Ewart C, Islam S. Caretaker expectations and the management of pediatric asthma in the inner city: A scale development study. *Soc Work Res*. 1998;22(1):51-59.

48. Bruzzese JM, Evans D, Mellins RB. Asthma self-management indices: Manual and instructions for the asthma: It's a family affair I project. *Unpublished report*. 2011.

49. Bruzzese J, Sheares BJ, Vincent EJ, et al. Effects of a school-based intervention for urban adolescents with asthma: A controlled trial. *Am J Respir Crit Care Med*. 2011;183:998--1006.

50. Aguilar-Fernandez AJ, Villa-Asensi JR, Castro-Codesal M, Almeria-Gil E, Gonzalez-Alvarez MI, Romero-Andujar F. Concordance between the piko - 1 portable device and pneumotachography in measuring PEF and FEV(1) in asthmatic children. *Allergol Immunopathol (Madr)*. 2009;37(5):244-248. doi: 10.1016/j.aller.2009.03.004 [doi].

51. Rothe T, Karrer W, Schindler C. Accuracy of the piko-1 pocket spirometer. *J Asthma*. 2012;49(1):45-50. doi: 10.3109/02770903.2011.643522 [doi].

52. Miller MR, Hankinson J, Brusasco V, et al. Standardisation of spirometry. *European Respiratory Journal*. 2005;26(2):319-338.

53. Rhee H, Belyea MJ, Brasch J. Family support and asthma outcomes in adolescents: Barriers to adherence as a mediator. *J Adolesc Health*. 2010.

54. Procidano ME, Heller K. Measures of perceived social support from friends and from family: Three validation studies. *Am J Community Psychol*. 1983;11(1):1-24.

55. Ozer EJ, Weinstein RS, Maslach C, Siegel D. Adolescent AIDS prevention in context: The impact of peer educator qualities and classroom environments on intervention efficacy. *Am J Community Psychol*. 1997;25(3):289-323.

56. Berridge BM, Crouchley R. *Multivariate Generalized Liner Mixed Models using R.* Boco Raton, FL: Chapman & Hall; 2011.

57. Hedeker D, Gibbons RD. *Longitudinal Data Analysis.* New York: Wiley; 2006.

58. Baron RM, Kenny DA. The moderator-mediator variable distinction in social psychological research: Conceptual, strategic and statistical considerations. *J Pers Soc Psychol*. 1986;51(6):1173-1182.

59. Preacher KJ, Hayes AF. Asympotic and resampling strategies for assessing and comparing indirect effects in multiple mediator models. *Behavior Research Methods*. 2008;40:879.

60. Chen DG, Peace KE. *Clinical Trial Data Analysis using R.* Boca Raton, FL: Chapman & Hall; 2010.

61. Federal Register. Payment policies under the physician fee schedule and other revisions to part B for CY 2012: Department of health and human services, centers for medicare & medicaid services. 2011;42 CFR Parts 410, 414, 415 and 419.

62. Finkler SA. The distinction between cost and charges. *Annals of Internal Medicine*. 1982;96(1):102-109.

63. Red Book. *Red Book (Red Book Drug Topics): Pharmacy's Fundamental Referene / the Physicians' Desk Reference (PDR).* ; 2010.

64. US Department of Labor, Bureau of Labor Statistics. Consumer price index.

65. Duan N, Manning WG, Morris CN, Newhouse JP. A comparison of alternative models for the demand for medical care. *J Bus Econ Stat*. 1983;1(2):115-126.
