# Supplementary material for: Long-term Effectiveness of a Peer-Led Asthma Self-management Program on Asthma Outcomes in Adolescents Living in Urban Areas: A Randomized Clinical Trial
Source: JAMA Netw Open. 2021 Dec 7;4(12):e2137492. doi: 10.1001/jamanetworkopen.2021.37492 (PMC8652603; doi:10.1001/jamanetworkopen.2021.37492)
Supplement: Supplement 2. — eTable 1. Means and Standard Deviations (SDs) of Study Variables by Treatment Group and Time Point (N=320) eTable 2. Longitudinal Effects of the Treatment on Individual Items of the Asthma Control Questionnaire in Adjusted Mean Differences (AMD) in Comparison to Baseline for Each Group After Controlling for Sex, Camp Season and Site (N=303, Intention to Treat Analysis) eFigure 1. Long-term Patterns of Quality of Life Subscales for Symptoms (A), Activity Limitations (B), and Emotional Functioning (C) by Group and Sex eFigure 2. Long-term Patterns of Asthma Control by Group and Sex eFigure 3. Long-term Patterns of Emotional Functioning for Bimonthly Contact Status (Any vs No) by Group [file jamanetwopen-e2137492-s002.pdf]

Supplemental Online Content

Rhee H, Love T, Wicks MN, et al. Long-term effectiveness of a peer-led asthma self-management program on asthma outcomes in adolescents living in urban areas: a randomized clinical trial. *JAMA Netw Open*. 2021;4(12):e2137492. doi:10.1001/jamanetworkopen.2021.37492

eTable 1. Means and Standard Deviations (SDs) of Study Variables by Treatment Group and Time Point (N=320)

eTable 2. Longitudinal Effects of the Treatment on Individual Items of the Asthma Control Questionnaire in Adjusted Mean Differences (AMD) in Comparison to Baseline for Each Group After Controlling for Sex, Camp Season and Site (N=303, Intention to Treat Analysis)

eFigure 1. Long-term Patterns of Quality of Life Subscales for Symptoms (A), Activity Limitations (B), and Emotional Functioning (C) by Group and Sex

eFigure 2. Long-term Patterns of Asthma Control by Group and Sex

eFigure 3. Long-term Patterns of Emotional Functioning for Bimonthly Contact Status (Any vs No) by Group

This supplemental material has been provided by the authors to give readers additional information about their work.

© 2021 Rhee H et al. *JAMA Network Open.*

eTable 1. Means and Standard Deviations (SDs) of Study Variables by Treatment Group and Time Point (N=320)

	**Enrollment** Mean, SD (n)		**Camp** Mean, SD (n)		**3-Month** Mean, SD (n)		**6-Month** Mean, SD (n)		**9-Month** Mean, SD (n)		**12-Month** Mean, SD (n)		**15-Month** Mean, SD (n)	
	PL	AL	PL	AL	PL	AL	PL	AL	PL	AL	PL	AL	PL	AL
**Quality of Life**														
Overall	5.21, 1.38 (168)	5.15, 1.31 (152)	5.6, 1.2 (134)	5.41, 1.26 (125)	5.7, 1.16 (145)	5.46, 1.23 (130)	5.7, 1.32 (133)	5.37, 1.28 (126)	5.72, 1.32 (133)	5.4, 1.4 (119)	5.89, 1.22 (133)	5.58, 1.36 (115)	5.93, 1.2 (139)	5.58, 1.36 (124)
Symptoms	5.02, 1.47 (168)	4.99, 1.4 (152)	5.44, 1.26 (134)	5.27, 1.33 (125)	5.57, 1.22 (145)	5.32, 1.29 (130)	5.57, 1.36 (132)	5.2, 1.37 (127)	5.59, 1.38 (133)	5.27, 1.47 (119)	5.8, 1.23 (133)	5.45, 1.42 (115)	5.85, 1.31 (139)	5.43, 1.42 (124)
Activity Limitations	5.19, 1.4 (168)	5.07, 1.37 (152)	5.62, 1.25 (134)	5.37, 1.36 (125)	5.62, 1.22 (145)	5.44, 1.24 (130)	5.6, 1.36 (133)	5.27, 1.34 (127)	5.67, 1.35 (133)	5.42, 1.4 (119)	5.76, 1.32 (133)	5.53, 1.37 (115)	5.82, 1.28 (139)	5.49, 1.39 (124)
Emotional Function	5.45, 1.45 (168)	5.39, 1.39 (152)	5.79, 1.32 (134)	5.61, 1.32 (125)	5.91, 1.23 (145)	5.66, 1.32 (130)	5.91, 1.39 (133)	5.6, 1.32 (126)	5.91, 1.38 (133)	5.56, 1.45 (119)	6.08, 1.28 (133)	5.76, 1.44 (115)	6.1, 1.15 (139)	5.81, 1.39 (124)
**Asthma Control**	1.48, 1.15 (168)	1.51, 1.1 (152)	1.12, 0.98 (134)	1.25, 1.05 (125)	1.1, 1.03 (145)	1.29, 1.04 (130)	1.12, 1.03 (132)	1.4, 1.08 (126)	1.04, 1.06 (134)	1.28, 1.1 (117)	0.95, 1 (133)	1.24, 1.18 (115)	0.92, 1.06 (139)	1.15, 1.01 (124)
**Asthma Management Index**														
Prevention	1.94 0.49 (168)	2.07 0.46 (152)	2.13 0.45 (133)	2.12 0.48 (125)	2.13 0.49 (144)	2.10 0.50 (129)	2.12 0.59 (132)	2.07 0.51 (125)	2.13 0.52 (134)	2.16 0.48 (117)	2.12 0.58 (132)	2.19 0.53 (115)	2.11 0.56 (136)	2.20 0.51 (124)
Management	5.27 1.67 (168)	5.39 1.39 (152)	5.62 1.35 (132)	5.65 1.30 (125)	5.66 1.18 (144)	5.52 1.31 (129)	5.59 1.46 (132)	5.48 1.44 (125)	5.53 1.51 (133)	5.58 1.38 (117)	5.53 1.52 (132)	5.52 1.52 (115)	5.67 1.31 (136)	5.51 1.54 (124)
Self-Efficacy	4.63, 0.82 (168)	4.55, 0.83 (152)	4.78, 0.95 (133)	4.64, 0.99 (125)	4.9, 0.79 (144)	4.72, 0.83 (128)	5.02, 0.79 (131)	4.78, 0.87 (126)	4.94, 0.87 (134)	4.75, 0.93 (117)	4.91, 0.93 (132)	4.75, 1.01 (115)	4.96, 0.85 (136)	4.79, 1.05 (124)
**Lung Function**														
FEV1	-	-	2.79, 0.76 (134)	2.73, 0.74 (125)	-	-	-	-	-	-	-	-	2.83, 0.77 (107)	2.71, 0.92 (98)
FVC	-	-	3.42, 0.85 (134)	3.38, 0.85 (125)	-	-	-	-	-	-	-	-	3.63, 0.84 (107)	3.61, 0.87 (98)
FEV1/FVC	-	-	0.82, 0.1 (134)	0.81, 0.1 (125)	-	-	-	-	-	-	-	-	0.78, 0.12 (107)	0.75, 0.18 (98)

Note: PL Peer-led (intervention group), AL Adult-led (control group); FEV1, FVC, FEV1/FVC were measured only two times at camp and 15-months; Spirometry was conducted only two times at camp and 15-months post-camp, hence the empty cells for other timepoints.

© 2021 Rhee H et al. *JAMA Network Open.*

eTable 2: Longitudinal Effects of the Treatment on Individual Items of the Asthma Control Questionnaire in Adjusted Mean Differences (AMD) in Comparison to Baseline for Each Group After Controlling for Sex, Camp Season and Site (N=303, Intention to Treat Analysis)

Variables	Group	Treatment effect size B (95%CI)	Treatment effect p-value	Treatment time interaction p-value	Adjusted Mean at Baseline	Post-camp AMD (95%CI)	p	3 mo AMD (95%CI)	p	6 mo, AMD (95%CI)	p	9 mo, AMD (95%CI)	p	12 mo, AMD (95%CI)	p	15 mo, AMD (95%CI)	p
Nighttime symptoms	Control Group	0	0.81	**0.04**	0.87	-0.16 (-0.40, 0.07)	0.17	-0.05 (-0.29, 0.20)	0.71	-0.07 (-0.32, 0.19)	0.60	-0.10 (-0.35, 0.16)	0.45	-0.16 (-0.41, 0.09)	0.20	-0.15 (-0.39, 0.10)	0.25
	Intervention Group	-0.02 (-0.22, 0.18)			1.17	-0.41 (-0.64, -0.18)	**0.001**	-0.48 (-0.71, -0.25)	**<0.001**	-0.55 (-0.80, -0.31)	**<0.001**	-0.41 (-0.65, -0.18)	**0.001**	-0.54 (-0.77, -0.31)	**<0.001**	-0.63 (-0.86, -0.39)	**<0.001**
Morning symptoms	Control Group	0	0.13	0.22	0.95	-0.11 (-0.35, 0.14)	0.39	-0.04 (-0.30, 0.21)	0.73	0.03 (-0.23, 0.28)	0.84	-0.10 (-0.36, 0.16)	0.44	-0.00 (-0.26, 0.25)	0.99	-0.13 (-0.39, 0.12)	0.30
	Intervention Group	-0.15 (-0.35, 0.05)			1.04	-0.34 (-0.57, -0.10)	**0.006**	-0.31 (-0.55, -0.07)	**0.01**	-0.35 (-0.60, -0.10)	**0.007**	-0.26 (-0.50, -0.02)	**0.04**	-0.38 (-0.62, -0.15)	**0.002**	-0.53 (-0.77, -0.29)	**<0.001**
Activity limitations	Control Group	0	**0.01**	0.42	1.34	-0.17 (-0.43, 0.09)	0.21	-0.30 (-0.57, -0.03)	**0.03**	-0.16 (-0.44, 0.12)	0.26	-0.40 (-0.68, -0.12)	**0.005**	-0.25 (-0.52, 0.03)	0.07	-0.53 (-0.80, -0.25)	**<0.001**
	Intervention Group	-0.27 (-0.47, -0.06)			1.20	-0.35 (-0.61, -0.10)	**0.007**	-0.41 (-0.67, -0.16)	**0.002**	-0.51 (-0.78, -0.24)	**<0.001**	-0.54 (-0.80, -0.28)	**<0.001**	-0.47 (-0.72, -0.21)	**<0.001**	-0.48 (-0.74, -0.21)	**<0.001**
Shortness of breath	Control Group	0	**0.03**	0.42	1.76	-0.26 (-0.55, 0.03)	0.07	-0.36 (-0.65, -0.07)	**0.01**	-0.34 (-0.64, -0.04)	**0.02**	-0.36 (-0.66, -0.06)	**0.01**	-0.39 (-0.68, -0.10)	**0.01**	-0.50 (-0.80, -0.21)	**0.001**
	Intervention Group	-0.26 (-0.50, -0.02)			1.75	-0.48 (-0.76, -0.21)	**0.001**	-0.60 (-0.87, -0.32)	**<0.001**	-0.65 (-0.94, -0.35)	**<0.001**	-0.75 (-1.03, -0.47)	**<0.001**	-0.83 (-1.10, -0.55)	**<0.001**	-0.73 (-1.01, -0.44)	**<0.001**
Wheezing	Control Group	0	**0.04**	0.31	1.63	-0.34 (-0.62, -0.07)	**0.01**	-0.31 (-0.59, -0.03)	**0.03**	-0.30 (-0.58, -0.01)	**0.04**	-0.40 (-0.68, -0.11)	**0.006**	-0.37 (-0.65, -0.09)	**0.01**	-0.39 (-0.67, -0.10)	**0.007**
	Intervention Group	-0.24 (-0.47, -0.00)			1.61	-0.42 (-0.68, -0.15)	**0.002**	-0.52 (-0.78, -0.25)	**<0.001**	-0.57 (-0.86, -0.29)	**<0.001**	-0.70 (-0.97, -0.43)	**<0.001**	-0.62 (-0.89, -0.36)	**<0.001**	-0.84 (-1.11, -0.57)	**<0.001**
SABA* use	Control Group	0	**0.03**	0.73	1.30	-0.10 (-0.32, 0.11)	0.33	-0.08 (-0.30, 0.14)	0.47	-0.12 (-0.34, 0.10)	0.29	-0.12 (-0.34, 0.11)	0.30	-0.07 (-0.29, 0.15)	0.52	-0.16 (-0.37, 0.06)	0.16
	Intervention Group	-0.19 (-0.36, -0.01)			1.25	-0.22 (-0.42, -0.01)	**0.04**	-0.16 (-0.36, 0.05)	0.13	-0.27 (-0.49, -0.05)	**0.02**	-0.32 (-0.53, -0.11)	**0.003**	-0.31 (-0.51, -0.10)	**0.003**	-0.35 (-0.56, -0.14)	**0.001**

*SABA Short acting beta agonist

© 2021 Rhee H et al. *JAMA Network Open.*

eFigure 1. Long-term Patterns of Quality of Life Subscales for Symptoms (A), Activity Limitations (B), and Emotional Functioning (C) by Group and Sex.

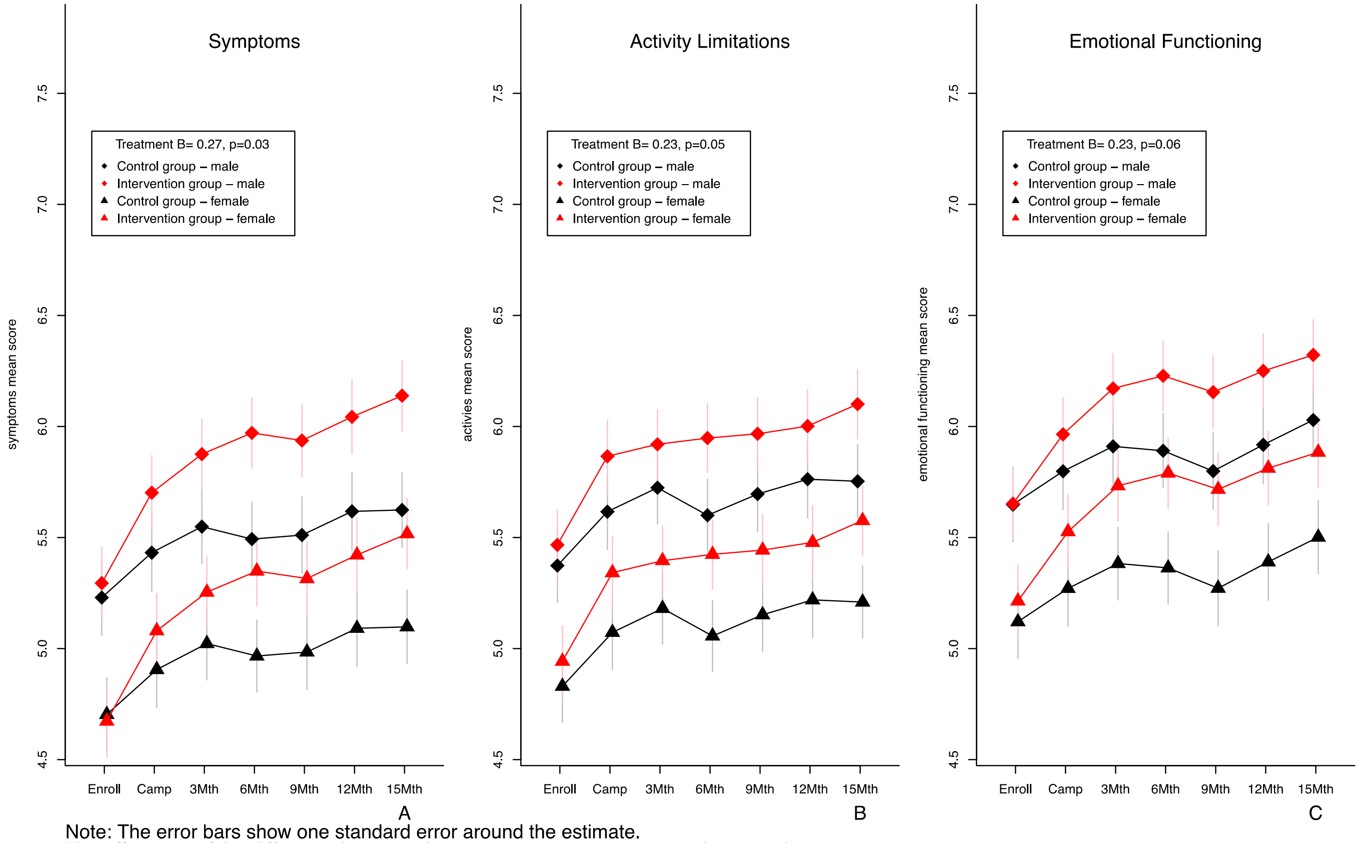

Note: The error bars show one standard error around the estimate.
The effect size of the difference between the treatment groups is given in the legend.

© 2021 Rhee H et al. *JAMA Network Open.*

eFigure 2. Long-term Patterns of Asthma Control by Group and Sex.

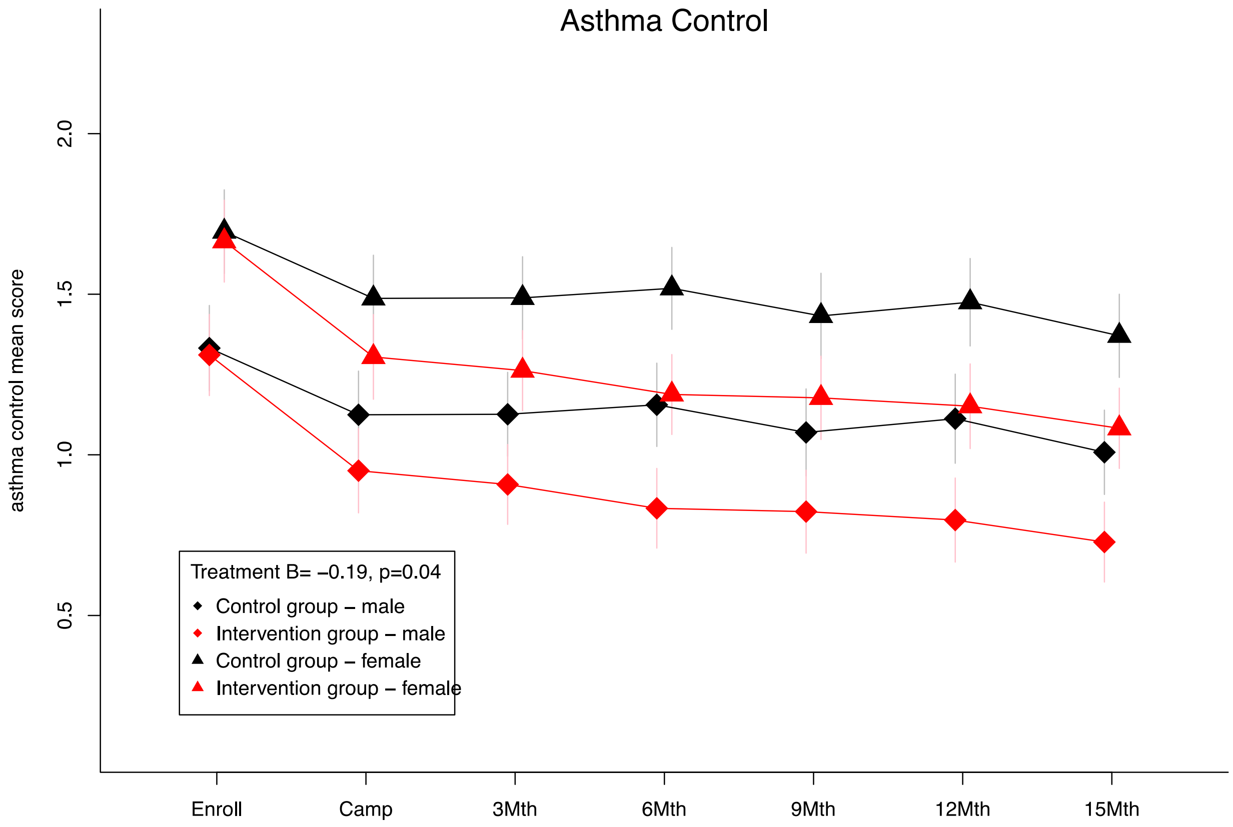

Note: The error bars show one standard error around the estimate.
The effect size of the difference between the treatment groups is given in the legend.

© 2021 Rhee H et al. *JAMA Network Open.*

eFigure 3. Long-term Patterns of Emotional Functioning for Bimonthly Contact Status (Any vs No) by Group.

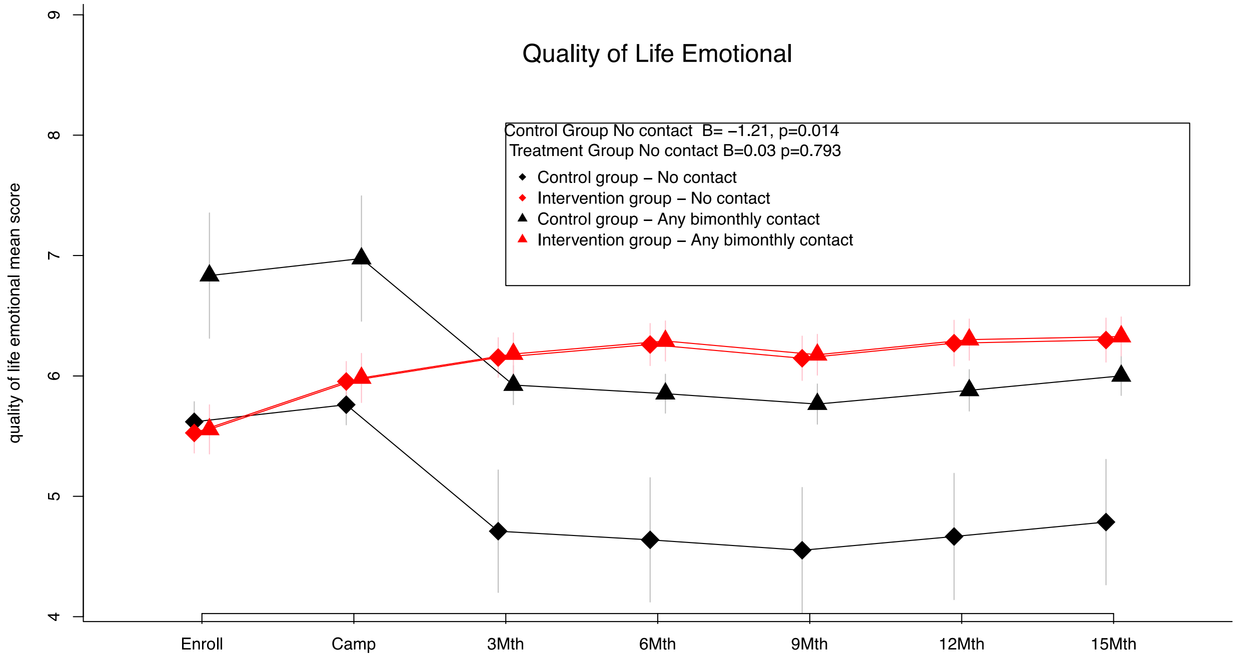

Note: The error bars show one standard error around the estimate. The legend shows the effect of having had any contact before each timepoint for each group. For the control group, there is a significant improvement (B=1.21, p=0.014) for those who have received any contact before the measurement. However, for the intervention group, there is no significant difference between those who have not yet been contacted and those who have (B=−0.03, p=0.793).

© 2021 Rhee H et al. *JAMA Network Open.*
