# Supplementary material for: Long-term Effectiveness of a Peer-Led Asthma Self-management Program on Asthma Outcomes in Adolescents Living in Urban Areas: A Randomized Clinical Trial
Source: JAMA Netw Open. 2021 Dec 7;4(12):e2137492. doi: 10.1001/jamanetworkopen.2021.37492 (PMC8652603; doi:10.1001/jamanetworkopen.2021.37492)
Supplement: Supplement 3. — Data Sharing Statement [file jamanetwopen-e2137492-s003.pdf]

# Data Sharing Statement

Rhee. Long-term Effectiveness of a Peer-Led Asthma Self-management Program on Asthma Outcomes in Adolescents Living in Urban Areas. *JAMA Netw Open*. Published December 07, 2021. doi:10.1001/jamanetworkopen.2021.37492

**Data**
**Data available:** Yes
**Data types:** Deidentified participant data, Data dictionary
**How to access data:** hyekyun.rhee@austin.utexas.edu
**When available:** With publication

**Supporting Documents**
**Document types:** Informed consent form
**How to access documents:** hyekyun.rhee@austin.utexas.edu
**When available:** With publication

**Additional Information**
**Who can access the data:** researchers whose proposed use of the data has been approved.
**Types of analyses:** for any purpose
**Mechanisms of data availability:** after approval of a proposal and with a signed data access agreement.
